# Characterization and Diversity of 243 Complete Human Papillomavirus Genomes in Cervical Swabs Using Next Generation Sequencing

**DOI:** 10.3390/v12121437

**Published:** 2020-12-14

**Authors:** Ardashel Latsuzbaia, Anke Wienecke-Baldacchino, Jessica Tapp, Marc Arbyn, Irma Karabegović, Zigui Chen, Marc Fischer, Friedrich Mühlschlegel, Steven Weyers, Pascale Pesch, Joël Mossong

**Affiliations:** 1 Epidemiology and Microbial Genomics, Laboratoire National de Santé, L-3555 Dudelange, Luxembourg; Anke.Wienecke-Baldacchino@lns.etat.lu (A.W.-B.); Jessica.TAPP@lns.etat.lu (J.T.); Irma.Karabegovic@lns.etat.lu (I.K.); Joel.Mossong@lns.etat.lu (J.M.); 2 Unit of Cancer Epidemiology, Belgian Cancer Centre, Sciensano, 1050 Brussels, Belgium; Marc.Arbyn@sciensano.be; 3 Department of Microbiology, The Chinese University of Hong Kong, Hong Kong, China; zigui.chen@cuhk.edu.hk; 4 Department of Medicine, Laboratoire National de Santé, L-3555 Dudelange, Luxembourg; fischer.cyto@lns.etat.lu; 5 Laboratoire National de Santé, L-3555 Dudelange, Luxembourg; Friedrich.MUeHLSCHLEGEL@lns.etat.lu; 6 Department of Obstetrics and Gynecology, Ghent University Hospital, 9000 Ghent, Belgium; Steven.Weyers@uzgent.be; 7 Planning Familial, L-1531 Luxembourg, Luxembourg; catherinechery@hotmail.com

**Keywords:** Anyplex II HPV28, cervical cancer, human papillomavirus, next generation sequencing, rolling-circle amplification

## Abstract

In recent years, next generation sequencing (NGS) technology has been widely used for the discovery of novel human papillomavirus (HPV) genotypes, variant characterization and genotyping. Here, we compared the analytical performance of NGS with a commercial PCR-based assay (Anyplex II HPV28) in cervical samples of 744 women. Overall, HPV positivity was 50.2% by the Anyplex and 45.5% by the NGS. With the NGS, we detected 25 genotypes covered by Anyplex and 41 additional genotypes. Agreement between the two methods for HPV positivity was 80.8% (kappa = 0.616) and 84.8% (kappa = 0.652) for 28 HPV genotypes and 14 high-risk genotypes, respectively. We recovered and characterized 243 complete HPV genomes from 153 samples spanning 40 different genotypes. According to phylogenetic analysis and pairwise distance, we identified novel lineages and sublineages of four high-risk and 16 low-risk genotypes. In total, 17 novel lineages and 14 novel sublineages were proposed, including novel lineages of HPV45, HPV52, HPV66 and a novel sublineage of HPV59. Our study provides important genomic insights on HPV types and lineages, where few complete genomes were publicly available.

## 1. Introduction

Human papillomavirus (HPV) is one of the most common sexually transmitted infections and the principal cause of cervical cancer [1]. HPVs belong to the *Papillomaviridae* family and are highly diverse. According to the International Committee on the Taxonomy of Viruses Study Group HPVs are classified in genera, species and types based on the *L1* open reading frame (ORF), being the most conserved region of the HPV genome [2]. Different HPV types share less than 90% of the *L1* gene nucleotide sequence [2]. HPV types with a difference in complete viral nucleotide sequence of 1% to 10% and of 0.5% to 1% have been further classified into lineages and sublineages denoted by letters and numbers, respectively [2,3,4]. Additionally, HPVs are classified according to their oncogenic potential into high-risk (hr) and low-risk (lr) genotypes [5,6].

Recent studies indicate that lineages of HPV16, 31 and 58 genotypes have different carcinogenic properties [3,7,8,9,10,11]. For example, sublineage D2 of HPV16 and lineages A/B of HPV31 are associated with an increased risk of cervical precancer compared to lineages A and C, respectively [8,9,12]. While the scientific community is mainly focusing on the characterization of hr-HPV, only a limited number of lr-HPV have been classified and characterized into lineages [13,14,15].

Modern technological advances have resulted in the development of more than 250 HPV detection and genotyping methods [16]. The Anyplex II HPV28 (Seegene, Seoul, Korea) is a widely used commercial PCR-based assay for the simultaneous detection and genotyping of 14 hr and 14 lr HPV types. This assay with high analytical performance has been clinically validated for cervical cancer screening and used in HPV vaccine surveillance studies [17,18,19,20].

While PCR-based HPV assays detect by design only a limited number of targeted genotypes, next generation sequencing (NGS) may be an alternative approach for universal HPV detection and characterization [16,21,22,23,24,25]. This technology facilitated the detection of novel HPV genotypes and variants [14,22,26] resulting in the identification of 222 HPV types and more than 100 lineages/sublineages [27,28]. Rolling-circle amplification (RCA) has been developed for the random amplification of circular DNA genomes, including viruses from the Papillomaviridae family [29]. It was first used for HPV amplification and sequencing by Rector et al. before the availability of NGS [30], and since then few studies have used this approach for investigating HPV [14,31,32]. The RCA technology allows unbiased random amplification of HPV genome without prior knowledge of the sequence and therefore has a great potential for improving characterization of both known and novel HPV genotypes [26,32,33].

In this study, we aimed to investigate and characterize HPV diversity circulating in healthy young women attending vaccine surveillance study in Luxembourg using NGS-RCA technology. In addition, we compared the analytical performance of the Anyplex II HPV28 assay and NGS using RCA for HPV detection and genotyping. To the best of our knowledge, this is the first large scale study to present HPV diversity using RCA-NGS in healthy women and compare the analytical performance of a PCR based genotyping assay with NGS-RCA.

## 2. Materials and Methods

A total of 744 cervical samples of women (median age: 22, range 18–43) participating in an HPV vaccination surveillance project were included in the study. More details on the study population and recruitment process have been published elsewhere [20]. Briefly, cervical samples were collected with a cervical broom by gynecologists or other physicians and placed in a vial containing PreservCyt medium of ThinPrep (Hologic, Inc., Bedford, MA, USA) [20,34].

### 2.1. DNA Extraction

DNA extraction from ThinPrep samples for sequencing and genotyping by the Anyplex II HPV28 was performed using the QIAamp DNA mini kit according to manufacturer’s instructions (Qiagen, Hilden, Germany) [35]. Prior to genotyping/sequencing DNA extracts were stored at −20 °C.

### 2.2. Anyplex II HPV28

Initially, HPV detection was performed by the Anyplex II HPV28, targeting the *L1* gene. The Anyplex assay can simultaneously identify and distinguish of 14 carcinogenic or hr-HPV types (16, 18, 31, 33, 35, 39, 45, 51, 52, 56, 58, 59, 66, 68), and 14 lr-HPV types (6, 11, 26, 40, 42, 43, 44, 53, 54, 61, 69, 70, 73, 82) in two sets. The viral load is defined as high (+++) if a positive signal occurs before 31 polymerase chain reaction (PCR) cycles, as medium (++) if a positive signal occurs within 31 to 40 PCR cycles, and low if a positive signal occurs after 41 PCR cycles [36].

Real time PCR was performed in two multiplex reactions on the CFX96 real-time thermocycler (Bio-Rad, Hercules, CA, USA). The human beta-globin housekeeping gene was amplified along with *L1* viral gene, as an internal positive control while water was used as a negative control. All reactions were performed according to the manufacturer’s instructions and the Seegene viewer software was used for data recording and interpretation [19].

### 2.3. Next Generation Sequencing

#### 2.3.1. Library Preparation

Total DNA extracts were enriched using rolling-circle amplification (RCA) technology with the TempliPhi 100 kit according to manufacturer’s instructions (GE Healthcare Life Sciences, New Jersey, NJ, USA) [30]. Libraries were prepared using Nextera XT DNA Library Prep Kits (Illumina Inc., San Diego, CA, USA) as recommended by manufacturer’s instructions followed by sequencing on Illumina MiniSeq Platform (Illumina Inc., San Diego, CA, USA). Up to 96 samples were multiplexed using standard Nextera DNA CD Illumina indices in one run (2 × 150 bp paired-end).

#### 2.3.2. Bioinformatic Analysis

##### Full Genome Assembly and Annotation

In order to assemble full HPV genome sequences, we followed two complementary approaches. For both approaches we performed an initial QC step, including visual inspection of FastQC report for each sample [37]. The strictness of applied quality criteria depended on the subsequent approach followed (Supplementary Figure S1A,B).

The first approach was to “blindly” assemble all reads from 744 samples (irrelevant of being HPV positive or negative) remaining after filtering against human, bacterial, plasmid and fungal reference sequences. The second approach was based on assemblies done on only dehumanized reads, which have been pre-mapped to all available HPV reference genome sequences. In both approaches mapping was performed with Bowtie2 [38] and de novo assembly with SPAdes (v. 3.13.0) [38,39]. Annotation of genomes was completed by means of an adapted version of VAPiD [40]. Detailed information on methods and annotation is provided in Supplementary Materials.

We obtained 75 identical genomes by both methods, 86 not fully identical, 42 only with blind assembly and 43 only with bowtie assembly. We selected 75 identical genomes, while not identical genomes were compared and further processed to allow assembly selection. All assemblies were checked by remapping fastq reads to assembled genomes and by aligning to the respective reference genomes. Of the total 246 genomes assessed, three (HPV42, HPV53, HPV56) were discarded from further analysis due to chimeric artefacts.

##### HPV Detection

For HPV detection and genotyping, quality controlled and dehumanized fastq-files from (trimmed and filtered against human genome as described above) were mapped with Bowtie2 to a reference set of 318 HPV sequences downloaded from PaVE (https://pave.niaid.nih.gov/, accessed in November 2018) and one novel genotype detected in our laboratory. Accession numbers of reference genomes are provided in Supplementary Table S1 [27].

Samples were considered positive if at least one concordantly mapping read pair was detected covering a minimum of 150 bp of the reference genome. To avoid possible mapping artefacts reads covering < 200 bp with the mutation rate > 0.03 (~4.5 variants on 150 bp read) were blasted against NCBI-based database (built on July 2019) and manually/visually checked. Artefacts were removed if the blast provided different results.

### 2.4. Phylogenetic Analysis and Lineage Classification

Complete HPV genomes obtained in this study (n = 243) were assessed to investigate HPV variant distribution in healthy women in Luxembourg. Complete genomes were linearized according to the respective reference sequence available in PaVE (https://pave.niaid.nih.gov/) [27] and aligned using MAFFT [41]. Each HPV isolate genome was classified into lineage or sublineage using the p-distance method and phylogenetic analysis with respective reference [3,4,13]. For the phylogenetic analysis the evolutionary history was inferred using RAxML method employing 1000 bootstrap values [42]. P-distance calculation was done in MEGA7 [43]. For HPV genomes with well-established lineages/sublineages we used the respective reference lineages and sublineages in PaVE and Chen et al. [13], whereas for HPVs with no established lineages/sublineages all complete genomes available in NCBI were downloaded for analysis (Supplementary Table S2). Phylogenetic trees were visualized in iTOL5.3 and Dendroscope 3 [44,45]. Plots were constructed in R (scripts available on request).

### 2.5. Statistical Analysis

Statistical analysis was performed using STATA 14 (College Station, TX, USA). We estimated HPV genotyping concordance by the percentage agreement and Cohen’s kappa (k). Cohen’s kappa values less than zero were classified as no agreement, 0.00–0.19 as poor, 0.20–0.39 as fair, 0.40–0.59 as moderate, 0.60–0.79 as good and 0.80–1.00 as excellent, as proposed in VALGENT [46,47]. Agreement, concordance and discordance were assessed restricting analysis to 28 genotypes included in the Anyplex II HPV28 assay and 729/744 samples with results available by both methods. Fully concordant samples were defined when all genotypes were detected by both Anyplex and NGS. Partially concordant samples were defined if at least one genotypes was shared by the two methods and discordant samples were defined as those where no genotypes were shared.

### 2.6. Data Access

Complete genomes generated during the study have been submitted to the European Nucleotide Archive (ENA) repository and are available under accession numbers LR861810, LR861811, LR861836–LR861981, LR861983, LR861984, LR861986, LR861987, LR861989, LR861990, LR861992–LR862007, LR862010, LR862011, LR862014, LR862015, LR862017, LR862018, LR862020–LR862086 (Supplementary Table S3). Sequence alignments are included in supplementary files. Accession numbers for genomes downloaded from GenBank are provided in Supplementary Tables S1 and S2.

### 2.7. Ethical Approval

The study was approved by the Comité National d’Ethique de Recherche (CNER # 201501/02 and 201909/01) and authorized by the Commission Nationale pour la Protection des Données (CNPD 288/2016).

## 3. Results

### 3.1. NGS and Anyplex II HPV 28

Overall, HPV positivity was 366/729 (50.2%) by Anyplex and 332/729 (45.5%) by NGS. From 28 genotypes covered by Anyplex, NGS detected 25 (all except HPV11, HPV26, HPV69) plus 41 additional genotypes in 171 (23.5%) samples, of which 43/171 (25.1%) were negative by Anyplex. Anyplex detected 146 genotypes in 97 samples negative by NGS. Overall, Anyplex detected 778 and NGS detected 888 type-specific infections, corresponding to an average of 1.1 and 1.2 genotypes per sample, respectively.

**Figure 1:**
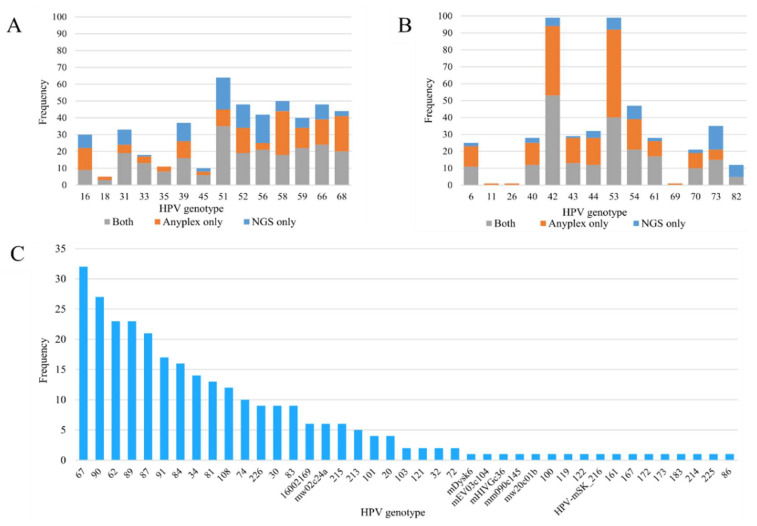
(**A**,**B**) Frequency distribution of human papillomavirus (HPV) genotypes detected by Anyplex II HPV28 and next-generation sequencing: (**A**) high-risk genotypes, (**B**) low-risk genotypes. Grey bars indicate genotypes detected by both methods, orange bars indicate genotypes detected by Anyplex II HPV28 only, and blue bars indicate genotypes detected by next-generation sequencing only. (**C**) Frequency distribution of HPV genotypes detected by next-generation sequencing only.

When considering only positive samples, the average number of genotypes detected per sample was 2.1 for Anyplex and 2.7 for NGS. Multiple genotypes were detected in 200/366 (54.6%, range 0–7) of the samples with Anyplex and 204/332 (61.4%, range 0–14) with NGS. Anyplex viral load was significantly associated with the count of reads mapping to HPV by NGS (*p* < 0.001) (Table 1). The top three most-frequently detected hr genotypes by Anyplex were HPV51 (5.8%), HPV58 (5.7%) and HPV68 (5.3%), while the top three genotypes by NGS were HPV51 (6.1%), HPV56 (4.3%) and HPV66 (3.7%) (Figure 1A). The most-frequently detected lr-HPV by both methods were HPV42 and HPV53 (Figure 1B). HPV67 (3.6%), HPV90 (3.0%), HPV62 (2.6%), HPV89 (2.5%) and HPV87 (2.3%) were the most-frequently observed genotypes detected by NGS (not targeted by Anyplex) (Figure 1C).

General agreement for overall HPV positivity of 28 genotypes between the two methods was 80.8% (kappa = 0.616) (good concordance). When restricting the analysis to medium and high viral load results (++/+++), agreement was higher (86.3%) with a kappa value of 0.712 (good concordance). Agreement for 14 hr HPV positivity was 84.8% with kappa value of 0.65 (good concordance). Similarly, when restricting analysis to medium and high viral load (++/+++) agreement for 14 hr-HPV was slightly higher (87.1%, kappa = 0.69) (good concordance) (Table 2). Type-specific agreement is reported in Supplementary Table S4. Among 398/729 (54.6%) HPV positive samples by either method, we observed 115/398 (28.9%) fully concordant samples down to genotype level, also considering infections with multiple genotypes.

### 3.2. HPV Variant Distribution and Lineage Classification

**Figure 2:**
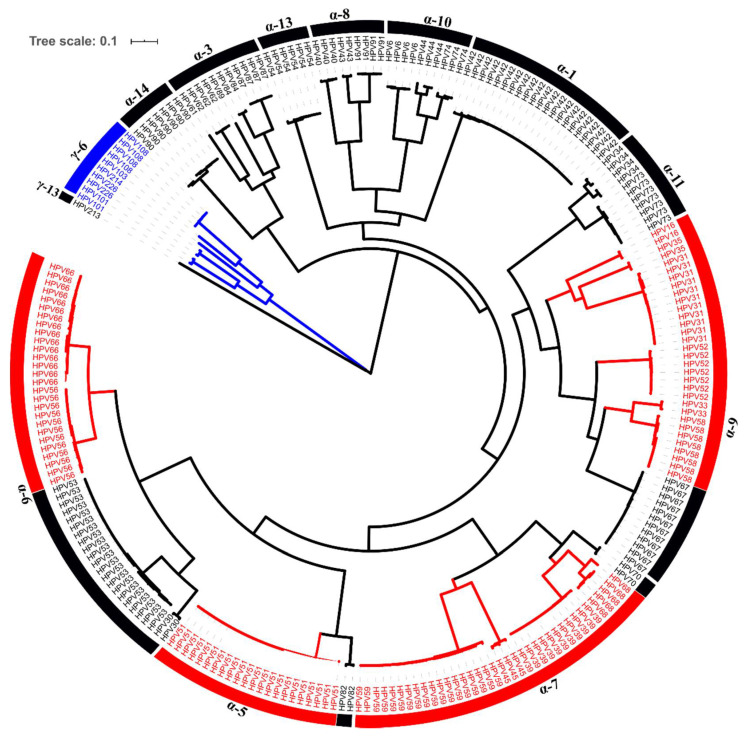
Phylogenetic tree of 243 HPV isolates. The maximum likelihood tree was constructed using RAxML based on complete HPV genomes. High-risk HPV types are shown in red. Gamma-6 species are colored in blue.

We obtained 232 complete alpha HPV and 11 gamma HPV genomes from 153 samples (Figure 2) spanning 40 different HPV types. The most-frequently obtained complete genomes were HPV51 (9.1%), HPV42 (8.6%), HPV53 (8.6%) and HPV59 (7.4%) (Table 3 and Table 4 and Supplementary Table S3). Phylogenetic trees were created for each isolate and topologies were evaluated to classify our genomes to the existing or novel lineages/sublineages as described before [4]. Reference HPV genomes were considered as the prototype sequence, which were always assigned as lineage A or sublineage A1 (Supplementary Table S2).

**Figure 3:**
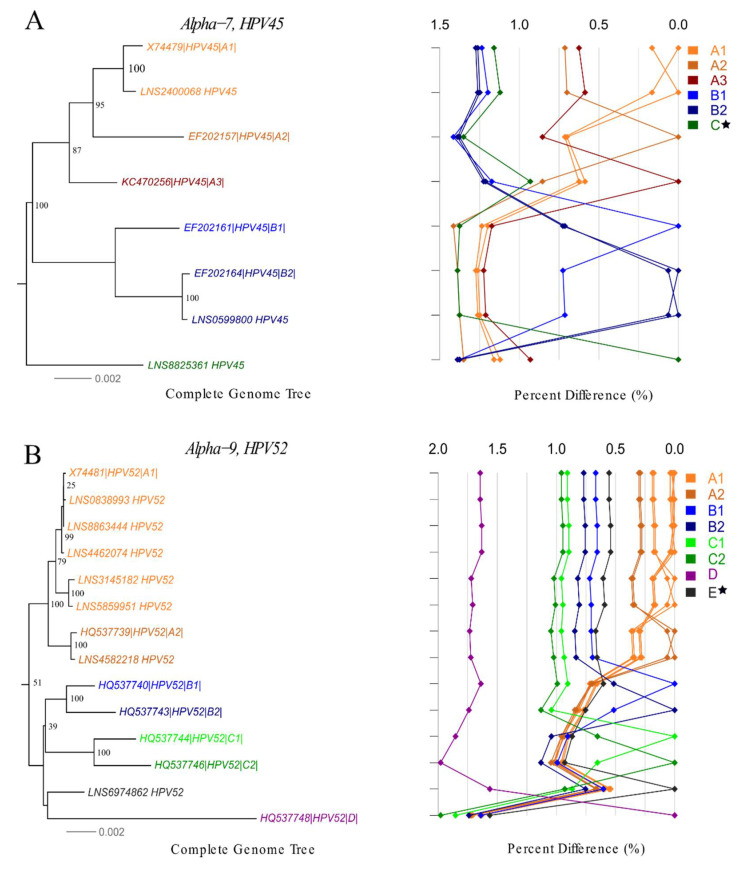

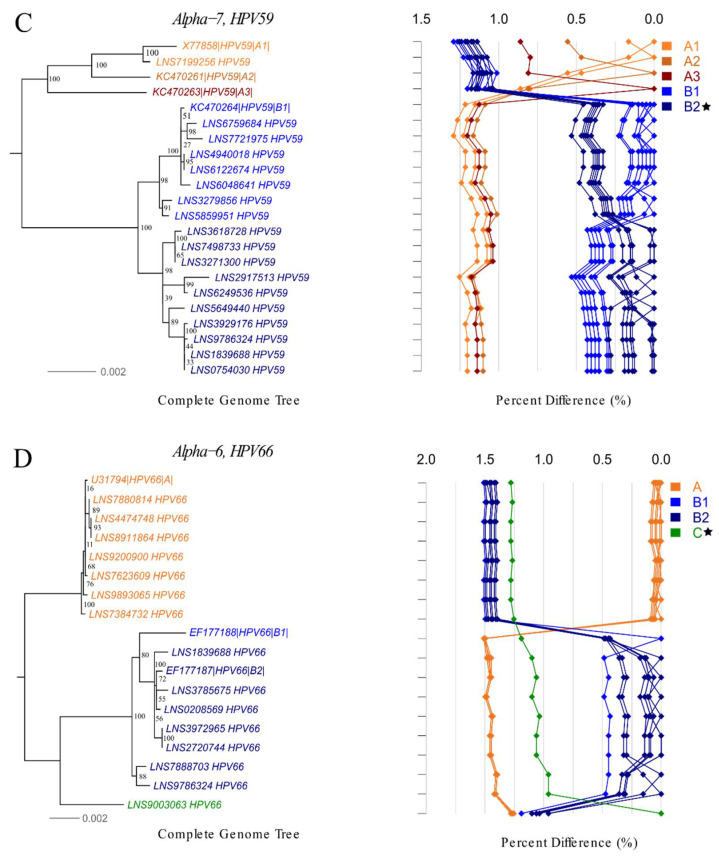
Phylogenetic trees and pairwise distance comparisons for novel lineages/sublineages of high-risk HPV complete genome variants. Phylogenetic trees were inferred from a global alignment of the complete genome nucleotide sequences of the variants from following HPV genotypes using RAxML: (**A**) HPV45, (**B**) HPV52, (**C**) HPV59, (**D**) HPV66. A star (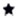) indicates novel lineage/sublineage. The phylogenetic topology is shown on the left, pairwise differences for each isolate are shown on the right. Values for each isolate are connected by lines of different colors to distinguish each lineage and sublineage. Genomes from our collection are indicated with LNS identification number.

**Figure 4:**
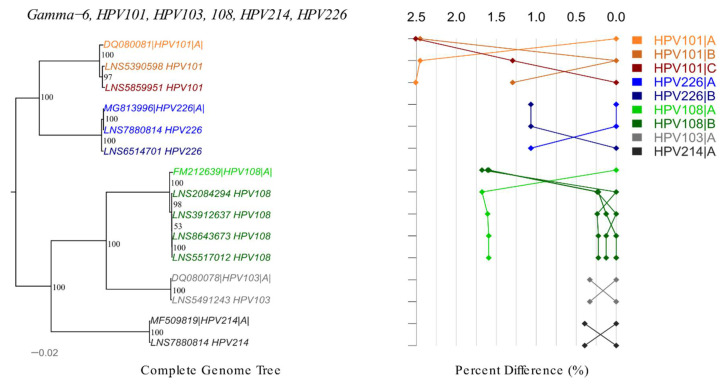
Phylogenetic tree and pairwise distance comparisons for gamma-6 species. Phylogenetic trees were inferred from a global alignment of the complete genome nucleotide sequences of the variants from following HPV genotypes using RAxML: HPV101, HPV103, HPV108, HPV214, HPV226. The phylogenetic topology is shown on the left, pairwise differences for each isolate are shown on the right. Values for each isolate are connected by lines of different colors to distinguish each lineage and sublineage. Genomes from our collection are indicated with LNS identification number.

Of a total 243 complete genomes, 49 (13 hr and 36 lr-HPV) were classified as belonging to 17 novel lineages and 14 novel sublineages of 20 HPV types including 4 hr and 16 lr-HPV genotypes (Table 3 and Table 4, Figure 3 and Figure 4 and Supplementary Figure S2). The remaining 194 complete genomes clustered with 46 existing lineages and 51 sublineages (Supplementary Figures S3 and S4).

#### 3.2.1. High-Risk HPV

In total, 118 complete hr-HPV genomes were assembled, spanning 24 existing lineages and 31 sublineages of 13 hr-HPV types (Supplementary Figure S3A–I). No complete genome of HPV18 could be obtained. We characterized novel lineages of HPV45, HPV52, HPV66 and a novel sublineage of HPV59 (Figure 3A–D). The novel lineage C of HPV45 differed by 0.93–1.35% and 1.38–1.39% from lineages A and B, respectively. The difference between the novel lineage E of HPV52 and other lineages ranged from 0.54% to 1.56%. Our analysis suggests that lineage B of HPV59 can be subdivided into sublineages B1 and B2. The pairwise distance between sublineages B1 and B2 varied from 0.27% to 0.53%. The novel lineage C of HPV66 differed by 1.25–1.41% and 0.96–1.32% from lineages A and B, respectively (Supplementary Table S5 and Figure 3A–D).

#### 3.2.2. Low-Risk HPV

In total, the 114 genomes obtained of 21 lr-HPV types clustered with 22 existing lineages and 20 sublineages (Supplementary Figure S4A–H). According to the pairwise distance and the phylogenetic analysis, we identified 10 novel lineages (HPV34, HPV42, HPV43, HPV54, HPV73, HPV74, HPV84, and HPV90) and 13 sublineages (HPV40, HPV42, HPV43, HPV61, HPV62, HPV67, HPV84, HPV90, and HPV91) (Supplementary Figure S2A–M). The distance between new lineages with reference variants varied between 0.86% and 5.12%, while distance between new sublineages with the nearest variant varied between 0.38% and 0.83% (Supplementary Table S5).

#### 3.2.3. Gamma HPV

We obtained 10 gamma-6 and one gamma-13 complete HPV genomes. In total, seven novel variants were identified including lineages A, B, C of HPV101 and lineages A, B of HPV108 and HPV226 (Figure 4). The difference amongst HPV101 lineages varied from 1.30% to 2.51%. The distance between A and B lineages was 1.59–1.68% for HPV108 and 1.07% for HPV226 (Supplementary Table S6). Variant of HPV213 gamma-13 genotype differed by 0.77% from reference genome.

## 4. Discussion

Our study showed a high diversity of human papillomavirus in women attending cervical cancer screening. We characterized 243 complete HPV genomes of 40 different genotypes and proposed the classification of novel lineages/sublineages for 4 hr-HPV and 16 lr-HPV genotypes. To the best of our knowledge, this is the first large scale study to present HPV diversity using unbiased NGS-RCA in healthy young women and comparing the analytical performance of a PCR-based genotyping assay with NGS-RCA.

Our findings suggest that the PCR-based the Anyplex assay is superior to the NGS in terms of sensitivity since only 16% of genotypes with low viral load by the Anyplex were concordantly identified by NGS. This is not surprising considering that Anyplex detection is based on type-specific primers, whereas RCA is a random amplification of circular DNA. Nevertheless, NGS detected an additional 110 genotypes compared to the Anyplex PCR. Further, our study suggests that concordance between the two methods increases with higher viral loads. In a recent clinical evaluation of the Anyplex assay, the detection of cervical intraepithelial neoplasia of grade 2 or worse (CIN2+) was associated with medium and high viral loads of the 14 high-risk types only, but not with low viral loads or low-risk genotypes [48]. In our study, only 3.5% participants were diagnosed with abnormal cytology, while we recovered 40% of complete genomes from these samples (Supplementary Table S7). As such, it would be important to validate the NGS approach in a clinical context using established protocol such as VALGENT [46].

In total, we detected 66 different HPV genotypes including 90 lineages/sublineages, of which 27 were novel and not previously described. Although, the diversity of hr-HPV genotypes has been well studied [4,13], we were able to identify novel variants of HPV45, HPV52, HPV59 and HPV66. Further, we proposed the classification of 10 novel lineages and 13 sublineages of lr-HPV which has been less studied. We were not able to obtain any complete HPV18 genomes (only five positive samples by both methods) as ~50% of the study population received HPV vaccine [20,49].

Several NGS-based approaches have been trialed, including RNA-baits, PCR-based and untargeted metagenomics [14,22,26,32,50,51,52]. Due to the high variability of HPV genomes across genera, species and even genotypes, designing primers or baits with acceptable sensitivity in a cost-effective manner is a major challenge. In our study, we found the untargeted rolling-circle amplification approach, initially implemented for HPV sequencing by Meiring [53], to be a very useful tool for characterizing HPV variants and detection. Identification of new variants could be important for adopting PCR-based assays, as we observed substantial discordance in HPV type-specific genotyping of some hr and lr HPVs.

Previous studies applied an RCA-based approach in immune-suppressed individuals, where high viral load would facilitate HPV detection and complete genome recovery. Pastrana et al. reported the discovery of 83 novel HPV types using RCA-NGS with the prior physical enrichment of viral DNA from immunodeficient patients [32]. This method was successfully used for HPV detection and characterization by researchers in South Africa and Brazil in HIV positive populations [14,53]. Results of the study by Wang et al. suggest that RCA-based sequencing is more accurate than the long-PCR template cloning and deep sequencing of HPV58 [54].

In our study, we obtained 243 HPV complete genomes, which increases the number of publicly available complete HPV genomes on GenBank (~2283 complete genomes on April 2020) by approximately 10%. Our dataset provides important genomic insights on HPV variants where currently only a few complete genomes were available, such as HPV42, HPV59, HPV66, HPV67, HPV90, and HPV91.

Interestingly, we also obtained 11 complete HPV genomes of gamma species, with a population prevalence of 3.7% in our population. Our findings are in line with the previous research which found gamma-6 species in cervical samples confirming their mucosal tropism [26,55,56,57,58]. Although gamma-6 pathogenicity is not well described, HPV108 could induce dysplasia in organotypic keratinocytes [57]. In our study, gamma-6 genotypes were present independently only in four samples, whereas other 23 samples were co-infected with other alpha HPVs. As gamma-6 HPV genotypes lack the *E6* oncogene, they may have a lower oncogenic potential [56].

One of the limitations of our study was the absence of negative and positive control during the sequencing, in order to account for the cross contamination and index hopping. Therefore, our detection criteria might produce some false positive results. Nevertheless, only 3% of the samples were defined as HPV positive by NGS with at least one concordantly mapping read pair to the reference genome. Moreover, when considering more stringent detection criteria for HPV positivity by NGS (mapping coverage of 1000 bp), the agreement between methods did not change significantly (Supplementary Table S8). The NGS detection threshold of concordant read pairs might have a higher impact on genotyping results, rather than HPV positivity. We accounted for potential PCR or sequencing errors during the assembly process by stringent quality controls and we excluded three genomes from the analysis due to the presence of chimeric contigs.

The major advantage of the NGS method is that it requires no prior knowledge of which HPV genotypes are present in the samples. Overall, we were able to detect 51 different HPV genotypes including 26, which are not detectable by the Anyplex assay. As the majority of these HPV types belong to IARC Group 3 (not classifiable as to its carcinogenicity to humans) [5], our findings currently do not have direct clinical implications in the context of cervical cancer screening. Nevertheless, this approach is certainly useful from a population and evolutionary biology perspective and may have potential to address new disease etiology in the future.

## Figures and Tables

**Table 1:** Proportion of genotypes detected by next generation sequencing (NGS) stratified by viral load as determined by Anyplex II HPV28.

Number of Genotypes Detected				Number of Reads per Genotype		Number of Reads per Sample	
Anyplex Viral Load	Anyplex	Concordant by NGS (%)	*p* Value	Mean	*p* Value	Mean	*p* Value
Low (+)	248	40 (16.1)	<0.001	1017	<0.001	905	<0.001
Medium (++)	344	223 (64.8)		1028		2390	
High (+++)	186	178 (95.7)		9994		18,162	

**Table 2:** Overall agreement for HPV detection between Anyplex II HPV28 and next-generation sequencing.

HPV Types ^1^	A+/NGS+ ^2^	A+/NGS− ^3^	A-/NGS+ ^4^	A-/NGS− ^5^	% Agreement	Kappa (se)	Interp-Retation ^6^
28 HPV types ^7^	258	108	32	331	80.8	0.616 (0.036)	G
28 HPV types (++/+++) ^8^	236	46	54	393	86.3	0.712 (0.037)	G
14 HPV types ^9^	179	71	40	439	84.8	0.652 (0.036)	G
14 HPV types (++/+++) ^10^	167	52	42	468	87.1	0.689 (0.037)	G

A, Anyplex; NGS, next generation sequencing; se, standard error. ^1^ Number of reads per sample restricted to 28 genotypes detectable by Anyplex. ^2^ A+/NGS+, positive with both methods. ^3^ A+/NGS-, Anyplex positive and NGS negative. ^4^ A-/NGS+, Anyplex negative and NGS positive. ^5^ A-/NGS-, negative with both methods. ^6^ Interpretation of the kappa values: P, poor; F, fair; M, moderate; G, good; E, excellent [47]. ^7^ Analysis restricted to 28 HPV types detectable by Anyplex. ^8^ Analysis restricted to 28 HPV types detectable by Anyplex, Anyplex positivity is restricted to medium or high viral load (++/+++). ^9^ Analysis is restricted to 14 high-risk HPV (HPV16,18,31,33,35,39,45,51,52,56,58,59, 66 and 68) [5]. ^10^ Analysis is restricted to 14 high-risk HPV, Anyplex positivity is restricted to viral load medium or high (++/+++).

**Table 3:** Classification of high-risk complete HPV genomes sequenced in our study.

Species	HPV Type	Genomes Sequenced	No. of Genomes on NCBI ^1^	No. of Genomes Used ^2^	Genome Size ^3^	GC Content (%)	Existing Lineage/Sublineage Sequenced	Novel Lineage/Sublineage Proposed
α-9	HPV16	2	566	10	7906–7907	36	A1	-
α-9	HPV31	11	29	7	7878–7920	37	A1, B2, C2–C3	-
α-9	HPV33	2	29	5	7833–7911	36	A1–A2	-
α-9	HPV35	2	30	2	7879–7880	36	A1	-
α-7	HPV39	11	22	3	7817–7885	40	A1–A2	-
α-7	HPV45	3	23	5	7849–7866	39	A1, B2	C
α-5	HPV51	22	31	6	7811–7815	39	A1–A3, B1	-
α-9	HPV52	7	93	7	7933–7962	38	A1–A2,	E
α-6	HPV56	12	13	3	7790–7866	37	A1–A2, B	-
α-9	HPV58	8	149	8	7823–7825	37–38	A2, B1–B2	-
α-7	HPV59	18	14	4	7898–7913	38	A1, B1	B2
α-6	HPV66	15	16	3	7816–7824	38	A, B2	C
α-7	HPV68	5	31	10	7814–7835	39–40	A1–A2, B, F2	-

GC Content, guanine-cytosine content. ^1^ Total number of complete genomes available on NCBI/GenBank. ^2^ Number of genomes downloaded from NCBI for the analysis. ^3^ Size of genomes sequenced in the study. For genotypes with established lineages/sublineages in PaVE only reference variants were used. For genotypes with no established lineages/sublineages in PaVE all complete genomes available in NCBI were used. See Supplementary Table S2 for the list of downloaded genomes.

**Table 4:** Classification of low-risk complete HPV genomes sequenced in our study.

Species	HPV Type	Genomes Sequenced	No. of Genomes on NCBI ^1^	No. of Genomes Used ^2^	Genome Size ^3^	GC Content (%)	Existing Lineage/Sublineage Sequenced	Novel Lineage/Sublineage Proposed
α-10	HPV6	4	214	4	8020–8032	40	B1, B3	–
α-6	HPV30	2	17	6	7843–7881	40	A2–A3	–
α-11	HPV34	4	17	5	7668–7788	37–38	A2, C2	D
α-8	HPV40	3	4	4	7905–7909	43	–	A2–A4
α-1	HPV42	21	8	8	7901–7920	39	A1, A2	A3, B, C
α-8	HPV43	2	2	2	7986–8007	40	–	B1–B2
α-10	HPV44	3	6	6	7822–7837	40–41	A, B	–
α-6	HPV53	21	29	7	7859–7864	40	A, C, D1–D2	–
α-13	HPV54	6	12	5	7760–7776	41	A2	D
α-3	HPV61	1	12	4	7989	46	–	A3
α-3	HPV62	3	4	4	8092–8092	45–46	A1	A2
α-9	HPV67	12	12	3	7806–7809	38	B1	B2
α-11	HPV73	7	16	3	7694–7716	36	A2, B	C
α-7	HPV70	2	10	2	7905–7911	40	A	–
α-10	HPV74	3	1	1	7893–7902	40–41	–	B, C
α-5	HPV82	2	22	10	7868–7874	40	A3, B1	–
α-3	HPV84	2	1	1	7956–7974	46	–	B1–B2
α-3	HPV87	4	4	4	7998–8001	45	A1	–
α-3	HPV89	1	4	4	8072	45	A2	–
α-14	HPV90	7	3		8016–8032	46	A1	A3, B
α-8	HPV91	4	1	1	7959–7959	40	–	A2
γ-6	HPV101	2	1	1	7258–7259	43	–	B, C
γ-6	HPV103	1	1	1	7263	41	–	–
γ-6	HPV108	4	1	1	7158–7158	42	–	B
γ-6	HPV214	1	1	1	7357	41	–	–
γ-6	HPV226	2	1	1	7313–7315	42		B
γ-13	HPV213	1	-		7096	39	–	–

GC Content, guanine-cytosine content. ^1^ Total number of complete genomes available on NCBI/GenBank. ^2^ Number of genomes downloaded from NCBI for the analysis. ^3^ Size of genomes sequenced in the study. For genotypes with established lineages/sublineages in PaVE only reference variants were used. For genotypes with no established lineages/sublineages in PaVE all complete genomes available in NCBI were used. See Supplementary Table S2 for the list of downloaded genomes.

## Supplementary Materials

The following are available online at https://www.mdpi.com/1999-4915/12/12/1437/s1, **Figure S1A.** Flow chart illustrating generation of blind assembly input data. Bar chart illustrating the generation of the blind assembly input data. The bar labels show the average single read count over 744 samples. For HG (Human Genome) we used genome version GRCh38. The average input size per sample for a blind genome assembly is 93,675 reads, which corresponds on average to ~14% of the raw sample size. **Figure S1B.** Flow chart illustrating generation of bowtie assembly input data. Bar chart illustrating the generation of the bowtie assembly input data. The lower graph shows the generation of the genome assembly input data only considering HPV positive samples. **Figure S2A–M.** Phylogenetic trees and pairwise distance comparisons for novel lineages/sublineages of low-risk HPV complete genome variants. Phylogenetic trees were inferred from a global alignment of the complete genome nucleotide sequences of the variants from following low risk HPV genotypes using RAxML: (A) HPV34, (B) HPV40, (C) HPV42, (D) HPV43, (E) HPV54, (F) HPV61, (G) HPV62, (H) HPV67, (I) HPV73, (J) HPV74, (K) HPV84, (L) HPV90, (M) HPV91. A star () indicates novel lineage/sublineage. The phylogenetic trees are shown on the left, pairwise differences for each isolate are shown on the right. Values for each isolate are connected by lines of different colors to distinguish each lineage and sublineage. Genomes from our collection are indicated with LNS identification number. **Figure S3A–H.** Phylogenetic trees and pairwise distance comparisons of high-risk HPV complete genome variants. Phylogenetic trees were inferred from a global alignment of the complete genome nucleotide sequences of the variants from following high risk HPV genotypes using RAxML: (A) HPV16, (B) HPV31, (C) HPV33, (D) HPV35, (E) HPV39, (F) HPV51, (G) HPV56, (H) HPV58, (I) 68. The phylogenetic trees are shown on the left, pairwise differences for each isolate are shown on the right. Values for each isolate are connected by lines of different colors to distinguish each lineage and sublineage. Genomes from our collection are indicated with LNS identification number. **Figure S4A–H.** Phylogenetic trees and pairwise distance comparisons of low-risk complete genome variants. Phylogenetic trees were inferred from a global alignment of the complete genome nucleotide sequences of the variants from following low risk HPV genotypes using RAxML: (A) HPV6, (B) HPV30, (C) HPV44, (D) HPV53, (E) HPV70, (F) HPV82, (G) HPV87, (H) HPV89. Phylogenetic trees are shown on the left, pairwise differences for each isolate are shown on the right. Values for each isolate are connected by lines of different colors to distinguish each lineage and sublineage. Genomes from our collection are indicated with LNS identification number.
