# Supplementary material for: Characterization and Diversity of 243 Complete Human Papillomavirus Genomes in Cervical Swabs Using Next Generation Sequencing
Source: Viruses. 2020 Dec 14;12(12):1437. doi: 10.3390/v12121437 (PMC7764970; doi:10.3390/v12121437)
Supplement: Supplementary file 1 [file viruses-12-01437-s001.zip › Supplementary material/Supplementary Fig S1A-B.pdf]

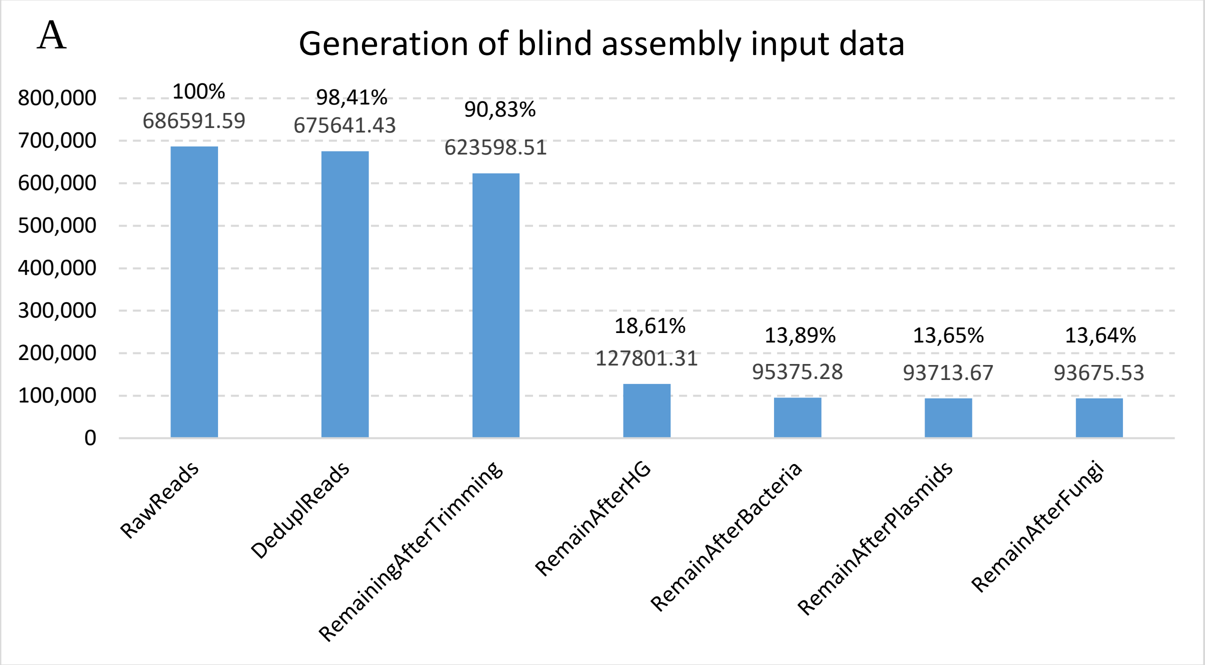
A
Generation of blind assembly input data
800,000
700,000
600,000
500,000
400,000
300,000
200,000
100,000
0
100%
686591.59
98,41%
675641.43
90,83%
623598.51
18,61%
127801.31
13,89%
95375.28
13,65%
93713.67
13,64%
93675.53
RawReads
DeduplReads
RemainingAfterTrimming
RemainAfterHG
RemainAfterBacteria
RemainAfterPlasmids
RemainAfterFungi

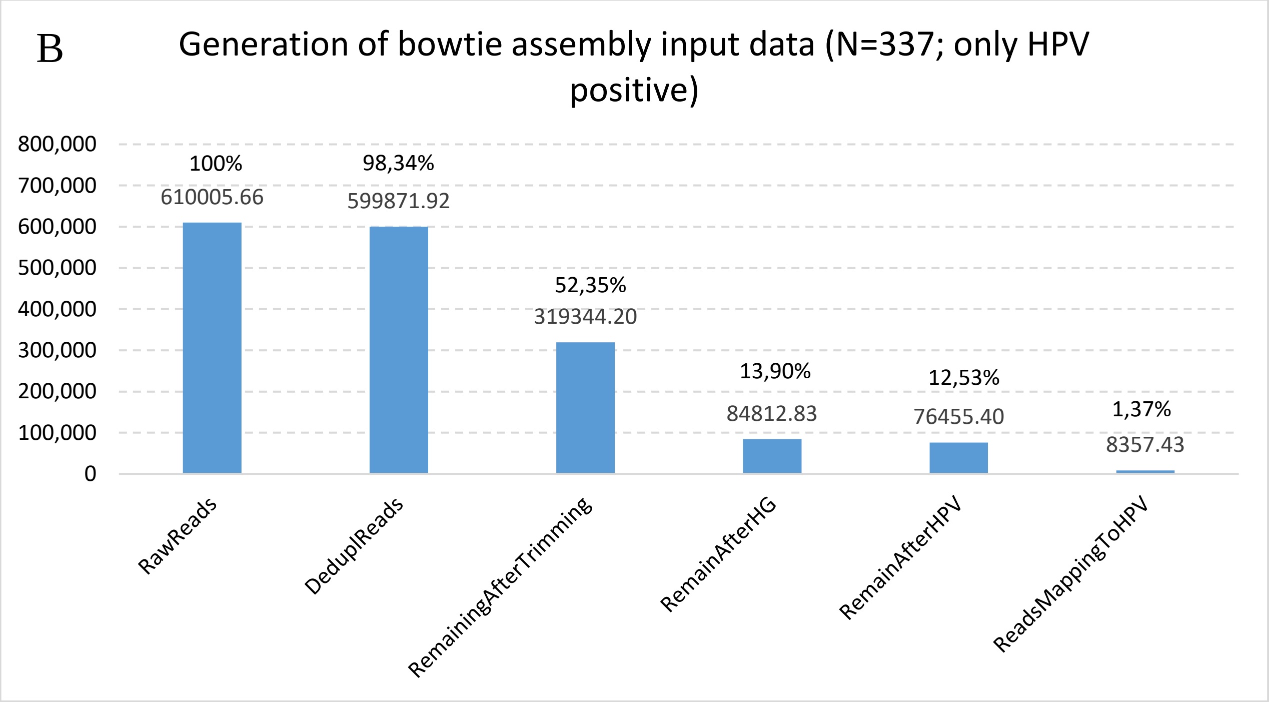
B
Generation of bowtie assembly input data (N=337; only HPV positive)
800,000
700,000
600,000
500,000
400,000
300,000
200,000
100,000
0
100%
610005.66
98,34%
599871.92
52,35%
319344.20
13,90%
84812.83
12,53%
76455.40
1,37%
8357.43
RawReads
DeduplReads
RemainingAfterTrimming
RemainAfterHG
RemainAfterHPV
ReadsMappingToHPV
