# Supplementary material for: Characterization and Diversity of 243 Complete Human Papillomavirus Genomes in Cervical Swabs Using Next Generation Sequencing
Source: Viruses. 2020 Dec 14;12(12):1437. doi: 10.3390/v12121437 (PMC7764970; doi:10.3390/v12121437)
Supplement: Supplementary file 1 [file viruses-12-01437-s001.zip › Supplementary material/Supplementary Fig S2A-M.pdf]

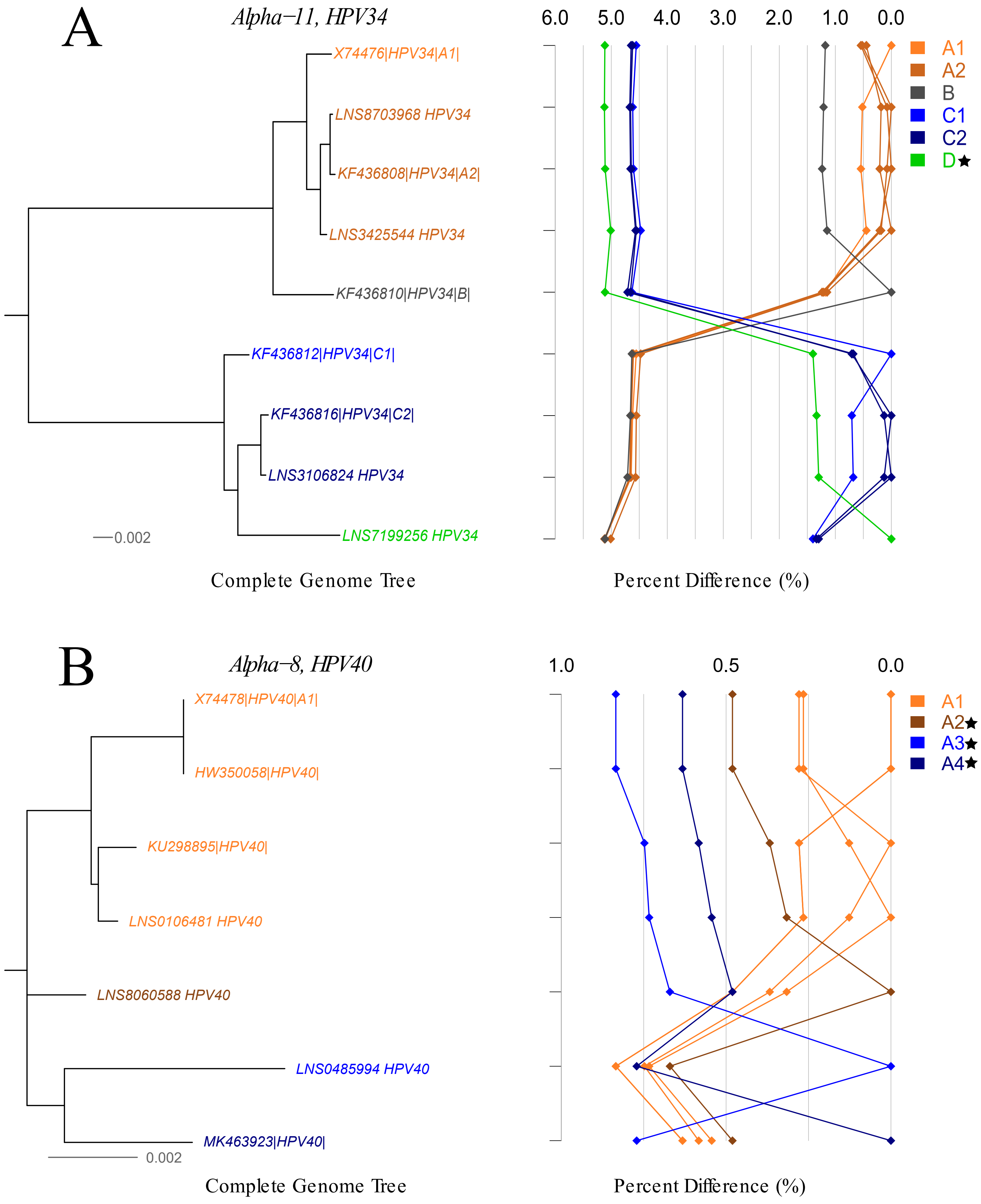

A
Alpha–11, HPV34
X74476|HPV34|A1|
LNS8703968 HPV34
KF436808|HPV34|A2|
LNS3425544 HPV34
KF436810|HPV34|B|
KF436812|HPV34|C1|
KF436816|HPV34|C2|
LNS3106824 HPV34
LNS7199256 HPV34
0.002
Complete Genome Tree
6.0 5.0 4.0 3.0 2.0 1.0 0.0
Percent Difference (%)
A1
A2
B
C1
C2
D★
B
Alpha–8, HPV40
X74478|HPV40|A1|
HW350058|HPV40|
KU298895|HPV40|
LNS0106481 HPV40
LNS8060588 HPV40
LNS0485994 HPV40
MK463923|HPV40|
0.002
Complete Genome Tree
1.0 0.5 0.0
Percent Difference (%)
A1
A2★
A3★
A4★

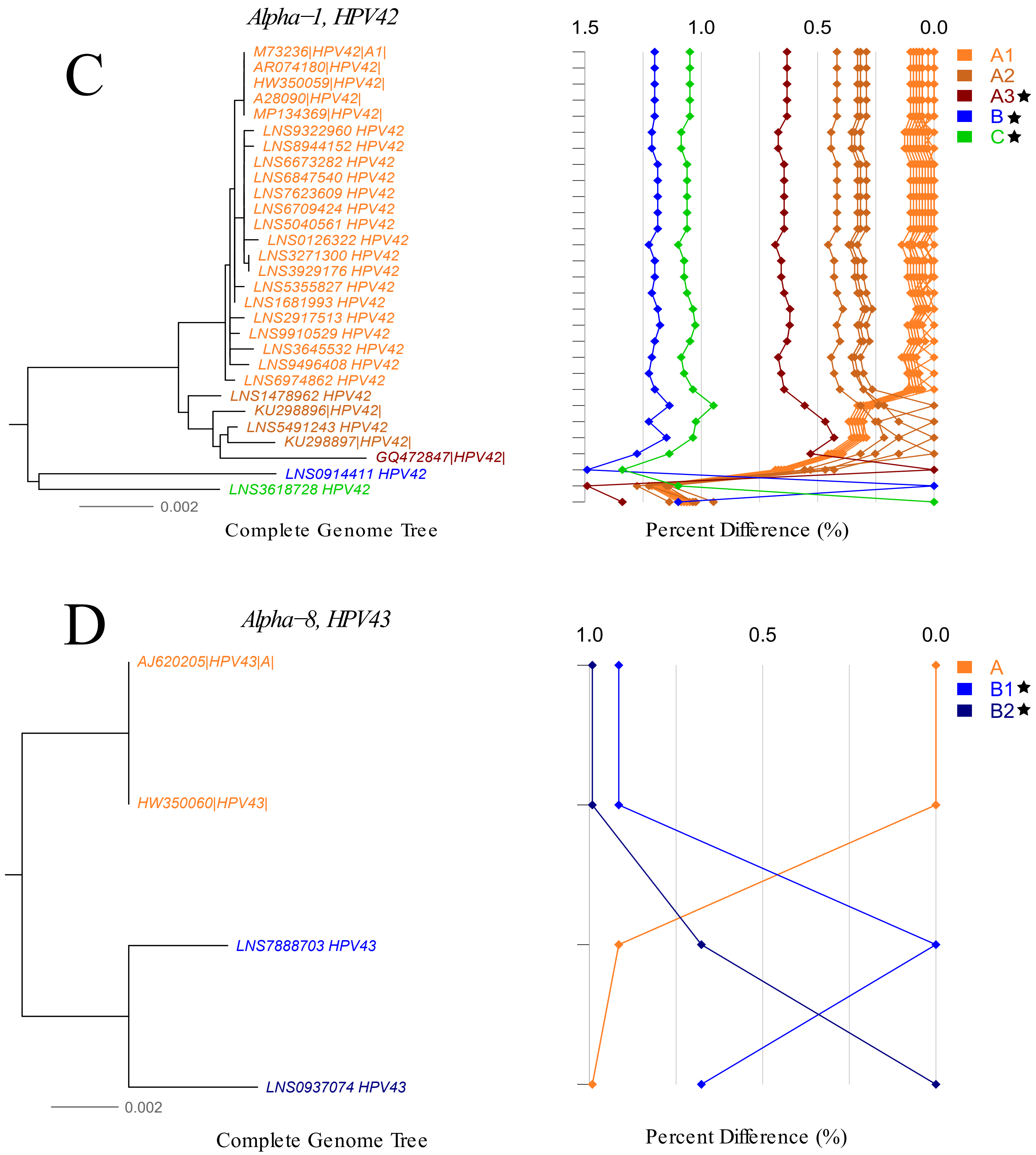
C
Alpha−1, HPV42
M73236|HPV42|A1|
AR074180|HPV42|
HW350059|HPV42|
A28090|HPV42|
MP134369|HPV42|
LNS9322960 HPV42
LNS8944152 HPV42
LNS6673282 HPV42
LNS6847540 HPV42
LNS7623609 HPV42
LNS6709424 HPV42
LNS5040561 HPV42
LNS0126322 HPV42
LNS3271300 HPV42
LNS3929176 HPV42
LNS5355827 HPV42
LNS1681993 HPV42
LNS2917513 HPV42
LNS9910529 HPV42
LNS3645532 HPV42
LNS9496408 HPV42
LNS6974862 HPV42
LNS1478962 HPV42
KU298896|HPV42|
LNS5491243 HPV42
KU298897|HPV42|
GQ472847|HPV42|
LNS0914411 HPV42
LNS3618728 HPV42
0.002
Complete Genome Tree
1.5
1.0
0.5
0.0
A1
A2
A3★
B★
C★
Percent Difference (%)
D
Alpha−8, HPV43
AJ620205|HPV43|A|
HW350060|HPV43|
LNS7888703 HPV43
LNS0937074 HPV43
0.002
Complete Genome Tree
1.0
0.5
0.0
A
B1★
B2★
Percent Difference (%)

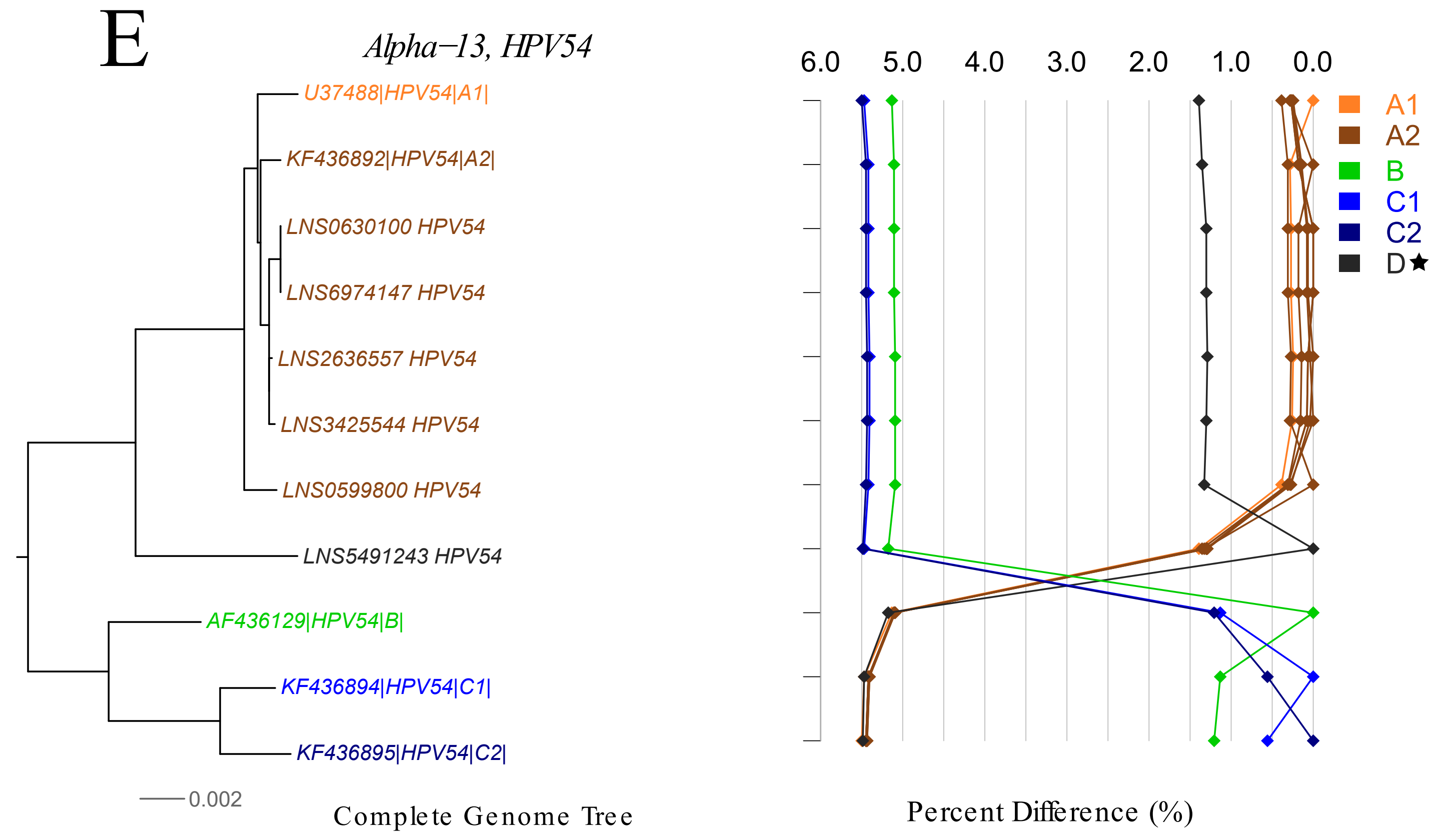
E
Alpha–13, HPV54
U37488|HPV54|A1|
KF436892|HPV54|A2|
LNS0630100 HPV54
LNS6974147 HPV54
LNS2636557 HPV54
LNS3425544 HPV54
LNS0599800 HPV54
LNS5491243 HPV54
AF436129|HPV54|B|
KF436894|HPV54|C1|
KF436895|HPV54|C2|
0.002
Complete Genome Tree
6.0
5.0
4.0
3.0
2.0
1.0
0.0
A1
A2
B
C1
C2
D★
Percent Difference (%)

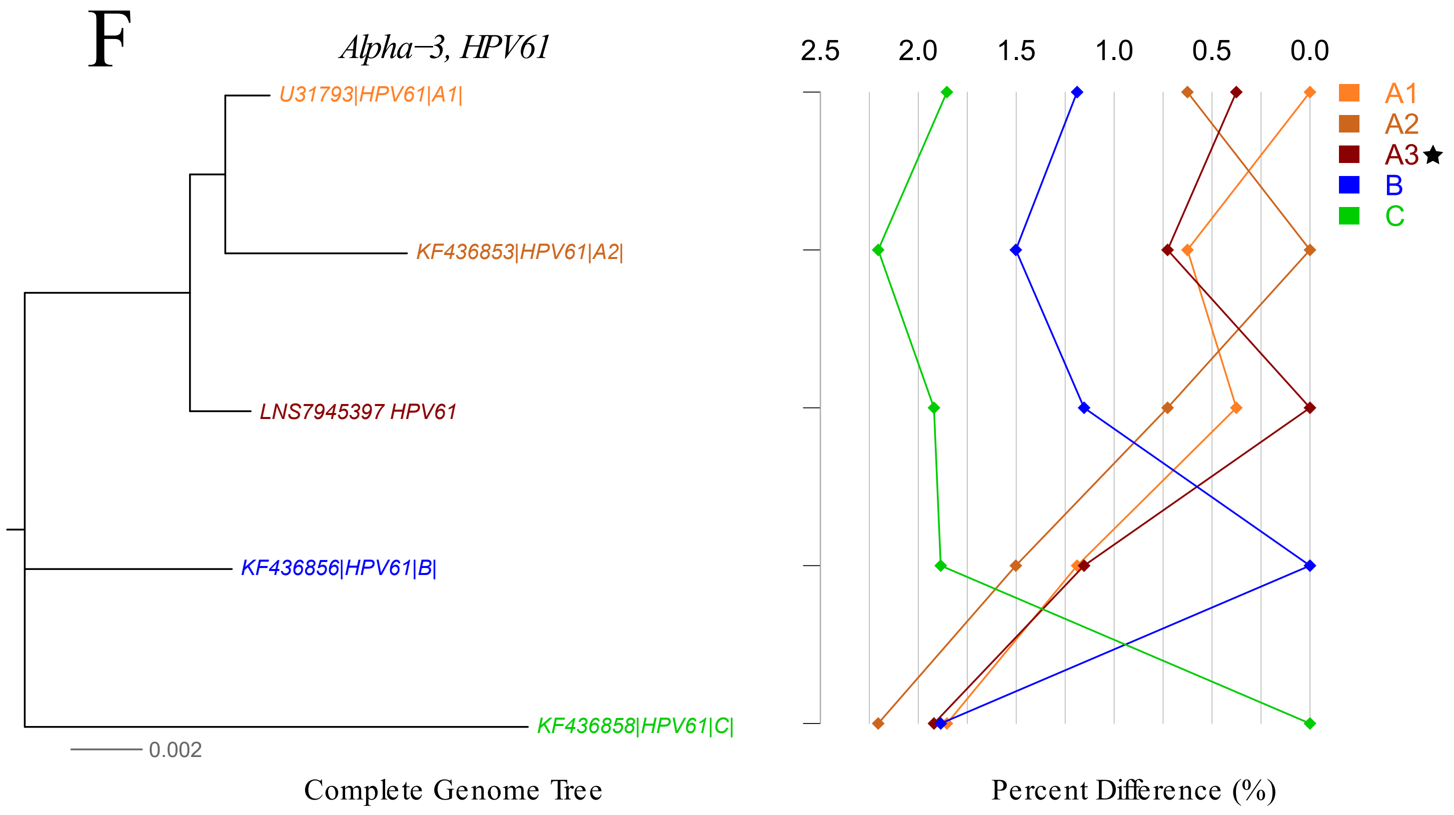
F
Alpha–3, HPV61
U31793|HPV61|A1|
KF436853|HPV61|A2|
LNS7945397 HPV61
KF436856|HPV61|B|
KF436858|HPV61|C|
0.002
Complete Genome Tree
2.5
2.0
1.5
1.0
0.5
0.0
A1
A2
A3★
B
C
Percent Difference (%)

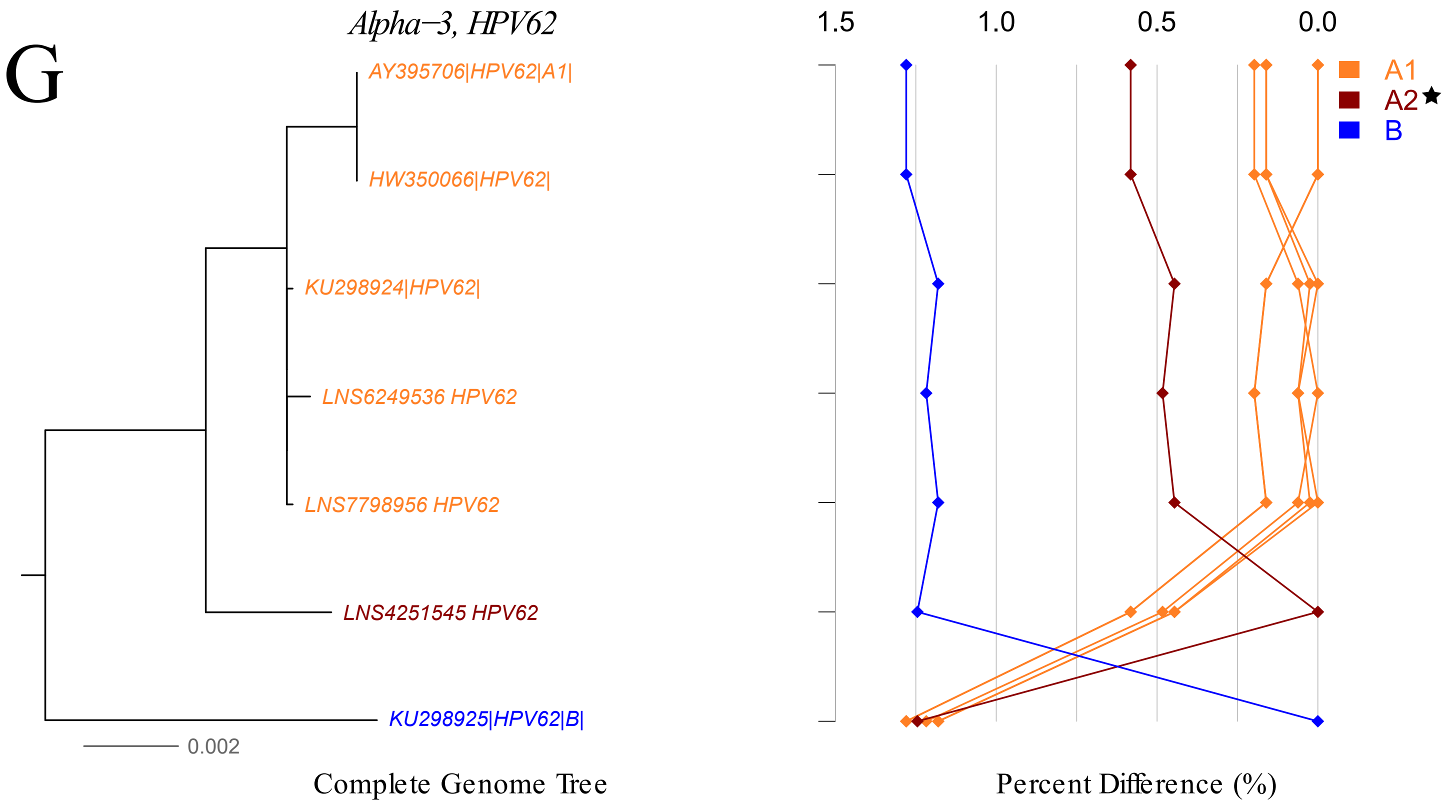
G
Alpha-3, HPV62
AY395706|HPV62|A1|
HW350066|HPV62|
KU298924|HPV62|
LNS6249536 HPV62
LNS7798956 HPV62
LNS4251545 HPV62
KU298925|HPV62|B|
0.002
Complete Genome Tree
1.5
1.0
0.5
0.0
A1
A2★
B
Percent Difference (%)

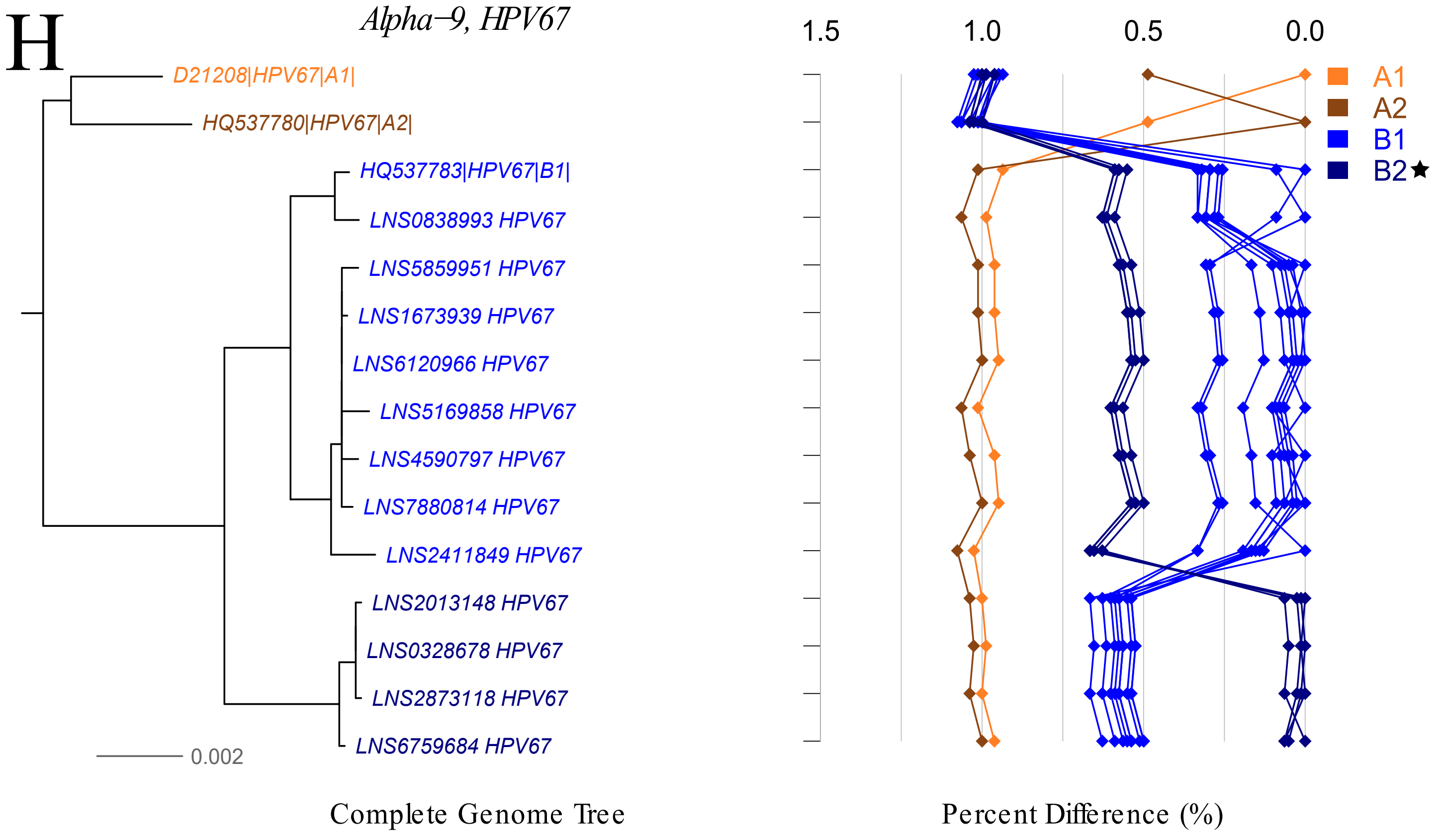
H
Alpha-9, HPV67
D21208|HPV67|A1|
HQ537780|HPV67|A2|
HQ537783|HPV67|B1|
LNS0838993 HPV67
LNS5859951 HPV67
LNS1673939 HPV67
LNS6120966 HPV67
LNS5169858 HPV67
LNS4590797 HPV67
LNS7880814 HPV67
LNS2411849 HPV67
LNS2013148 HPV67
LNS0328678 HPV67
LNS2873118 HPV67
LNS6759684 HPV67
0.002
Complete Genome Tree
1.5
1.0
0.5
0.0
A1
A2
B1
B2★
Percent Difference (%)

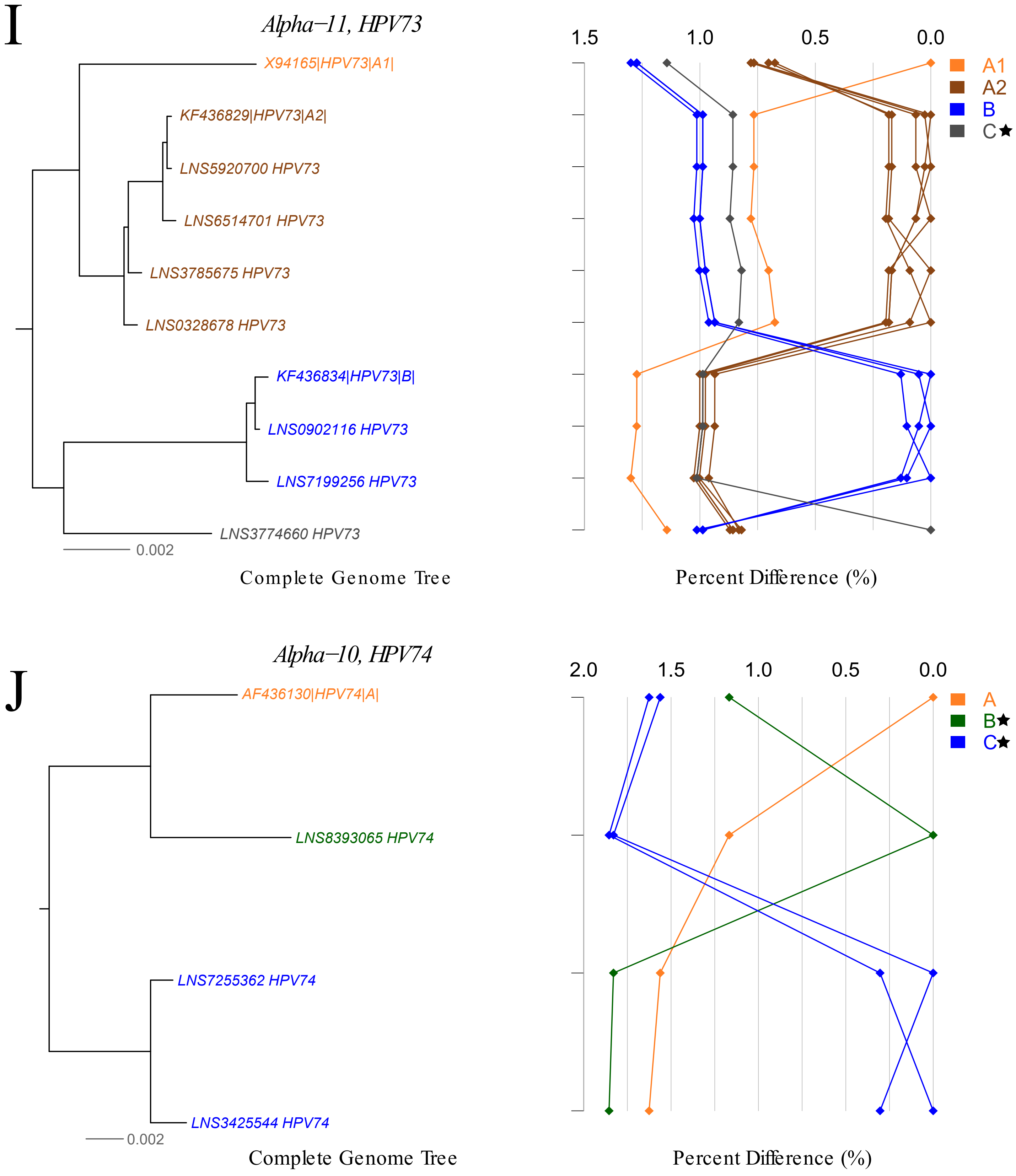
I
Alpha−11, HPV73
X94165|HPV73|A1|
KF436829|HPV73|A2|
LNS5920700 HPV73
LNS6514701 HPV73
LNS3785675 HPV73
LNS0328678 HPV73
KF436834|HPV73|B|
LNS0902116 HPV73
LNS7199256 HPV73
LNS3774660 HPV73
0.002
Complete Genome Tree
1.5
1.0
0.5
0.0
A1
A2
B
C★
Percent Difference (%)
J
Alpha−10, HPV74
AF436130|HPV74|A|
LNS8393065 HPV74
LNS7255362 HPV74
LNS3425544 HPV74
0.002
Complete Genome Tree
2.0
1.5
1.0
0.5
0.0
A
B★
C★
Percent Difference (%)

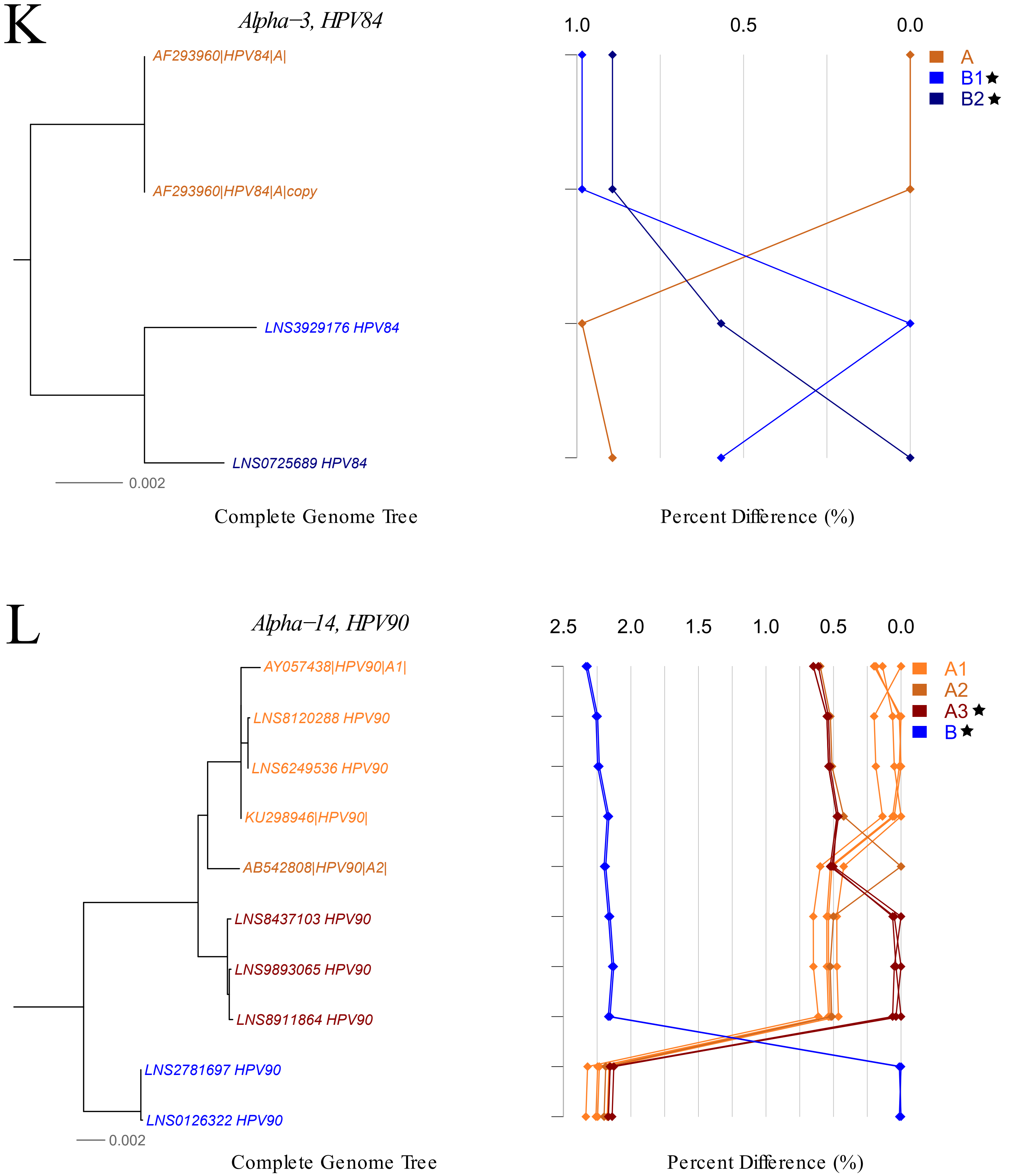
K
Alpha–3, HPV84
AF293960|HPV84|A|
AF293960|HPV84|A|copy
LNS3929176 HPV84
LNS0725689 HPV84
0.002
Complete Genome Tree
1.0
0.5
0.0
A
B1★
B2★
Percent Difference (%)
L
Alpha–14, HPV90
AY057438|HPV90|A1|
LNS8120288 HPV90
LNS6249536 HPV90
KU298946|HPV90|
AB542808|HPV90|A2|
LNS8437103 HPV90
LNS9893065 HPV90
LNS8911864 HPV90
LNS2781697 HPV90
LNS0126322 HPV90
0.002
Complete Genome Tree
2.5
2.0
1.5
1.0
0.5
0.0
A1
A2
A3★
B★
Percent Difference (%)

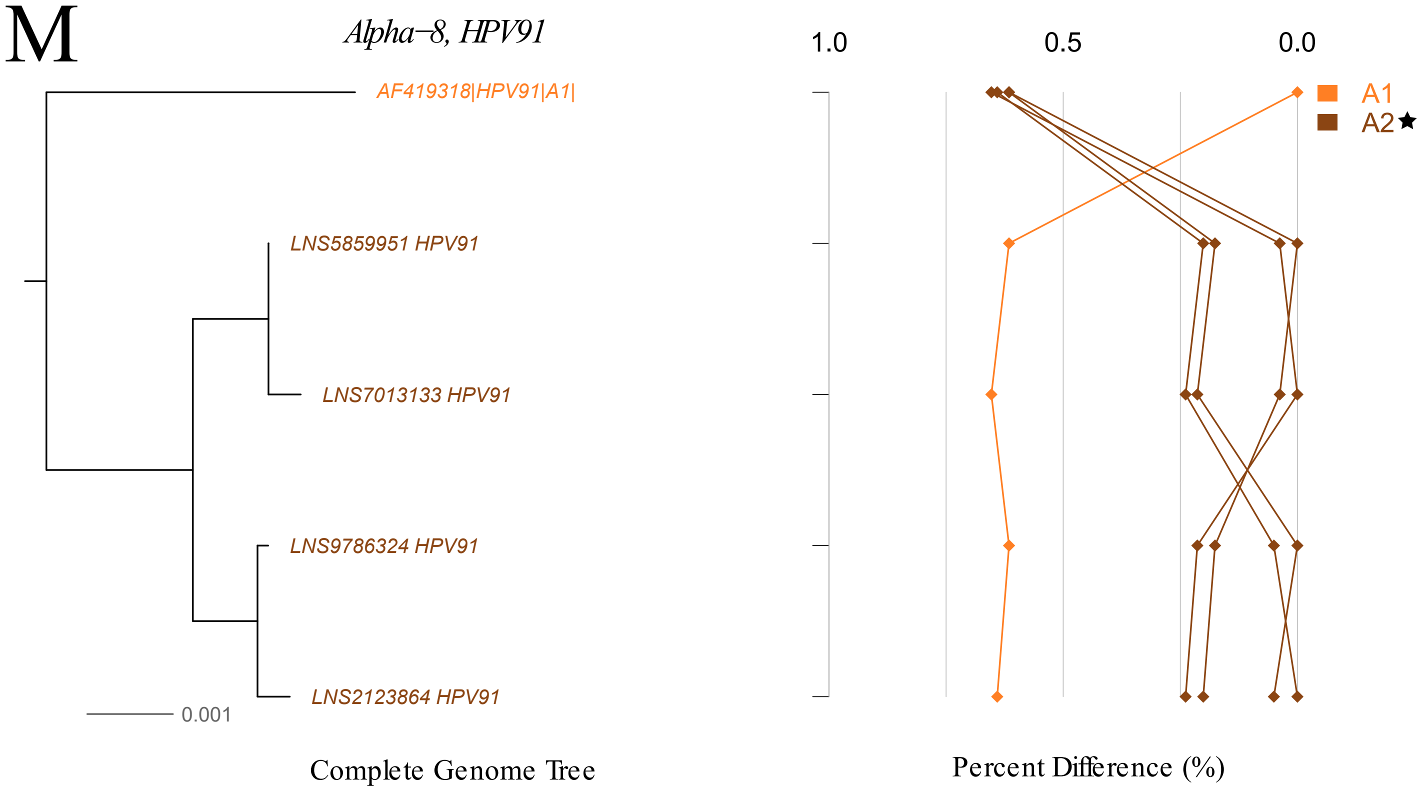
M
Alpha-8, HPV91
AF419318|HPV91|A1|
LNS5859951 HPV91
LNS7013133 HPV91
LNS9786324 HPV91
LNS2123864 HPV91
0.001
Complete Genome Tree
1.0
0.5
0.0
A1
A2★
Percent Difference (%)
