# Supplementary material for: Characterization and Diversity of 243 Complete Human Papillomavirus Genomes in Cervical Swabs Using Next Generation Sequencing
Source: Viruses. 2020 Dec 14;12(12):1437. doi: 10.3390/v12121437 (PMC7764970; doi:10.3390/v12121437)
Supplement: Supplementary file 1 [file viruses-12-01437-s001.zip › Supplementary material/Supplementary Fig S3A-I.pdf]

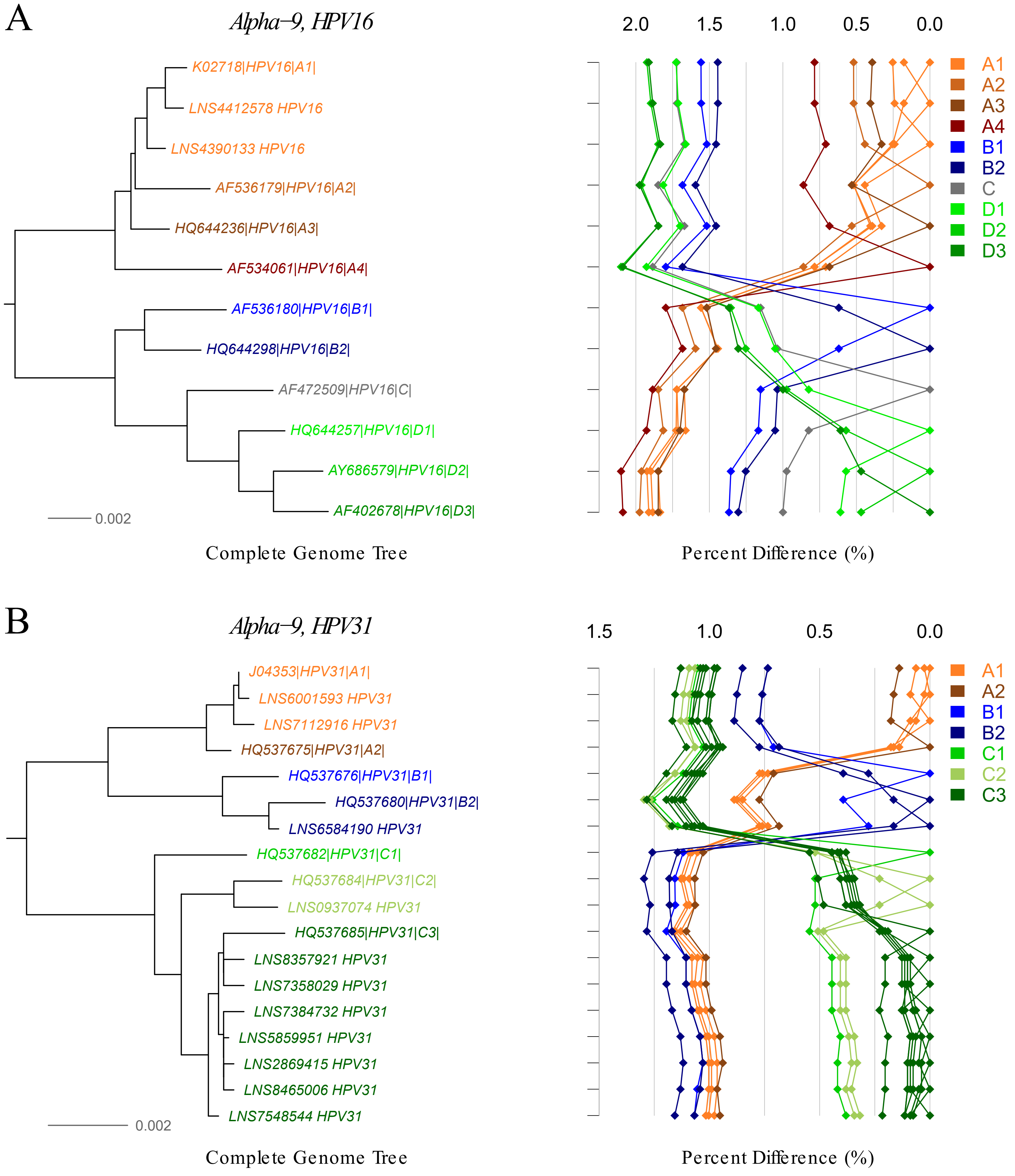
A
Alpha-9, HPV16
K02718|HPV16|A1|
LNS4412578 HPV16
LNS4390133 HPV16
AF536179|HPV16|A2|
HQ644236|HPV16|A3|
AF534061|HPV16|A4|
AF536180|HPV16|B1|
HQ644298|HPV16|B2|
AF472509|HPV16|C|
HQ644257|HPV16|D1|
AY686579|HPV16|D2|
AF402678|HPV16|D3|
0.002
Complete Genome Tree
2.0 1.5 1.0 0.5 0.0
A1 A2 A3 A4 B1 B2 C D1 D2 D3
Percent Difference (%)
B
Alpha-9, HPV31
J04353|HPV31|A1|
LNS6001593 HPV31
LNS7112916 HPV31
HQ537675|HPV31|A2|
HQ537676|HPV31|B1|
HQ537680|HPV31|B2|
LNS6584190 HPV31
HQ537682|HPV31|C1|
HQ537684|HPV31|C2|
LNS0937074 HPV31
HQ537685|HPV31|C3|
LNS8357921 HPV31
LNS7358029 HPV31
LNS7384732 HPV31
LNS5859951 HPV31
LNS2869415 HPV31
LNS8465006 HPV31
LNS7548544 HPV31
0.002
Complete Genome Tree
1.5 1.0 0.5 0.0
A1 A2 B1 B2 C1 C2 C3
Percent Difference (%)

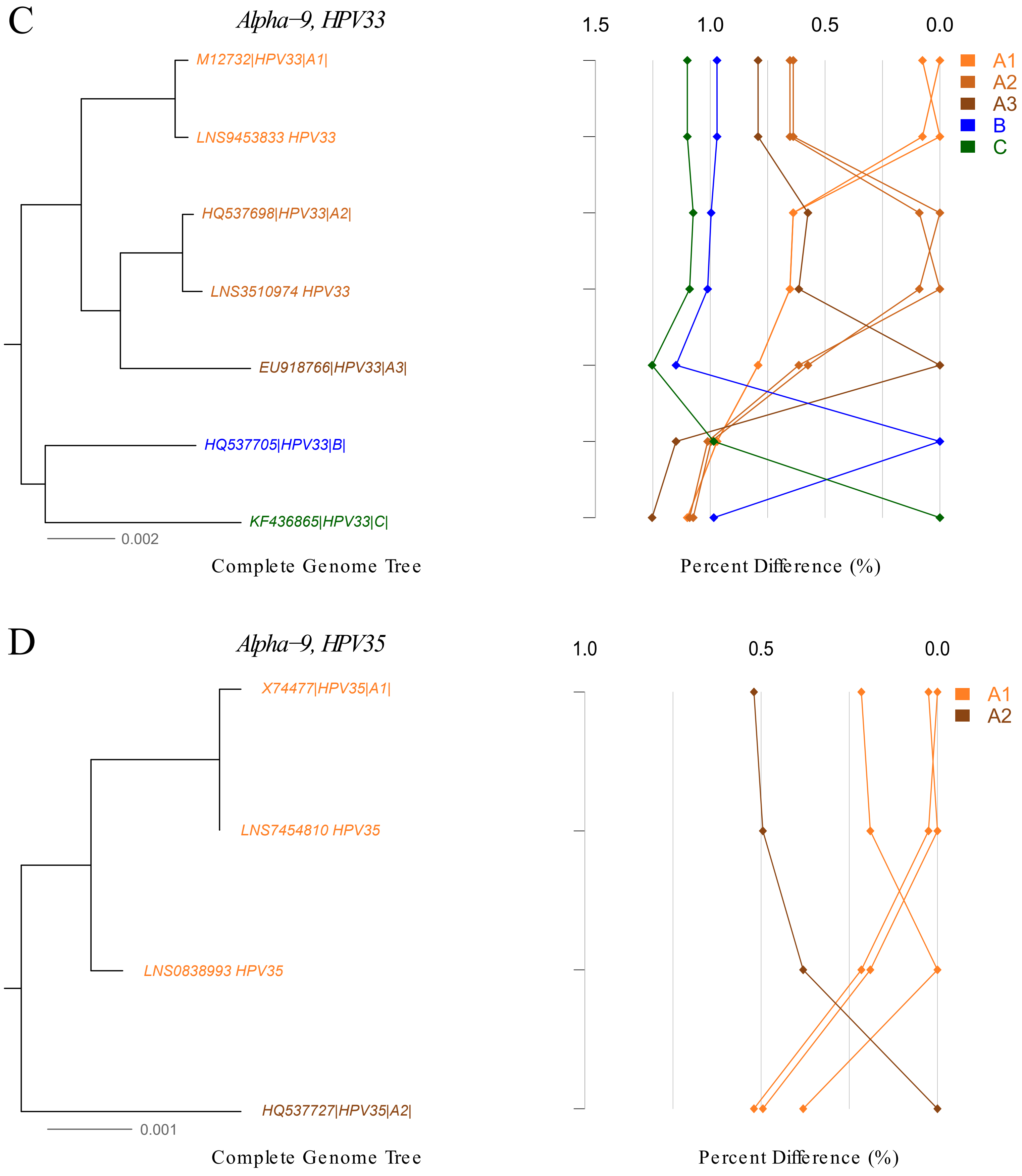
C
Alpha-9, HPV33
M12732|HPV33|A1|
LNS9453833 HPV33
HQ537698|HPV33|A2|
LNS3510974 HPV33
EU918766|HPV33|A3|
HQ537705|HPV33|B|
KF436865|HPV33|C|
0.002
Complete Genome Tree
1.5
1.0
0.5
0.0
A1
A2
A3
B
C
Percent Difference (%)
D
Alpha-9, HPV35
X74477|HPV35|A1|
LNS7454810 HPV35
LNS0838993 HPV35
HQ537727|HPV35|A2|
0.001
Complete Genome Tree
1.0
0.5
0.0
A1
A2
Percent Difference (%)

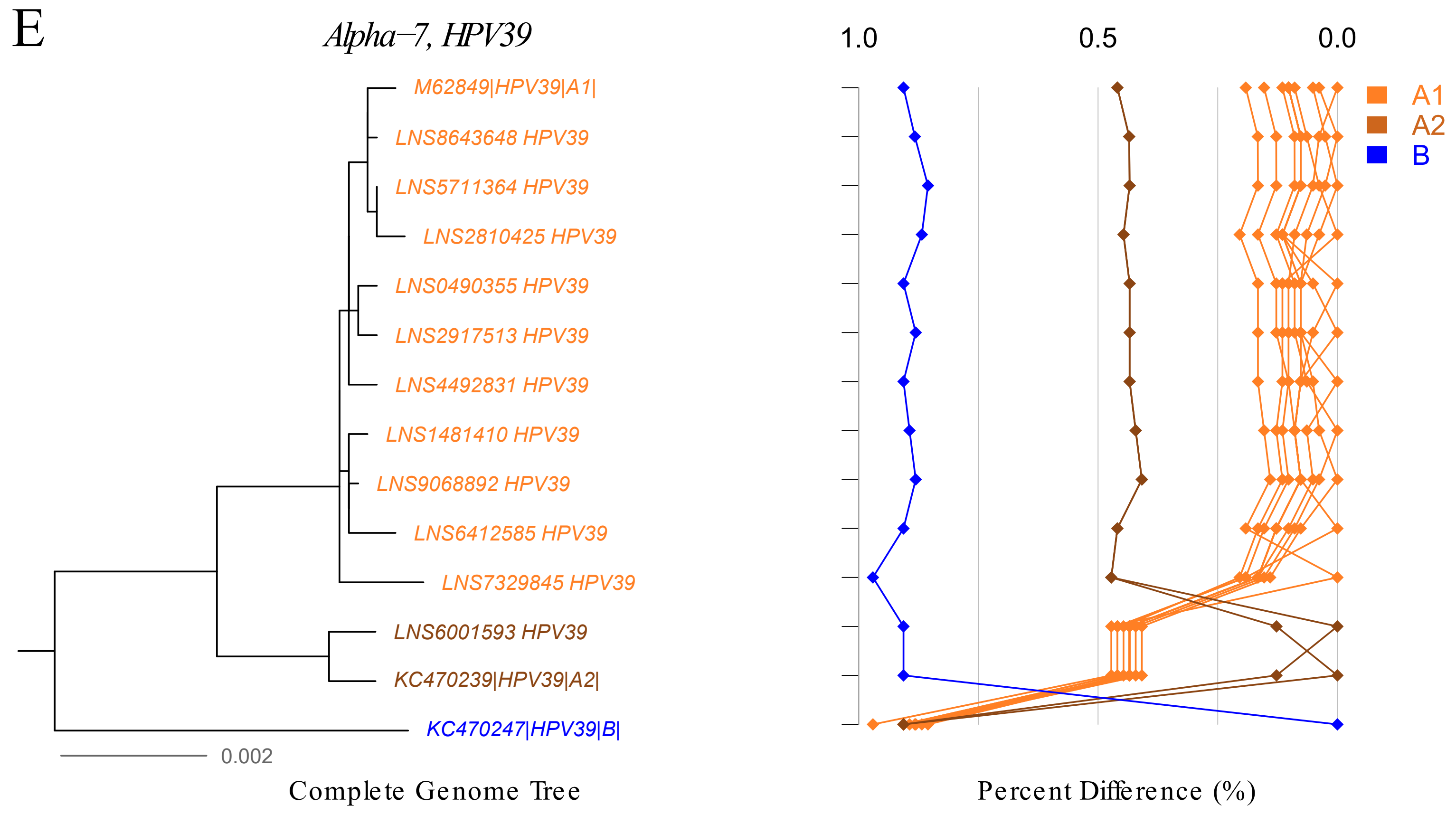
E
Alpha-7, HPV39
M62849|HPV39|A1|
LNS8643648 HPV39
LNS5711364 HPV39
LNS2810425 HPV39
LNS0490355 HPV39
LNS2917513 HPV39
LNS4492831 HPV39
LNS1481410 HPV39
LNS9068892 HPV39
LNS6412585 HPV39
LNS7329845 HPV39
LNS6001593 HPV39
KC470239|HPV39|A2|
KC470247|HPV39|B|
0.002
Complete Genome Tree
1.0
0.5
0.0
A1
A2
B
Percent Difference (%)

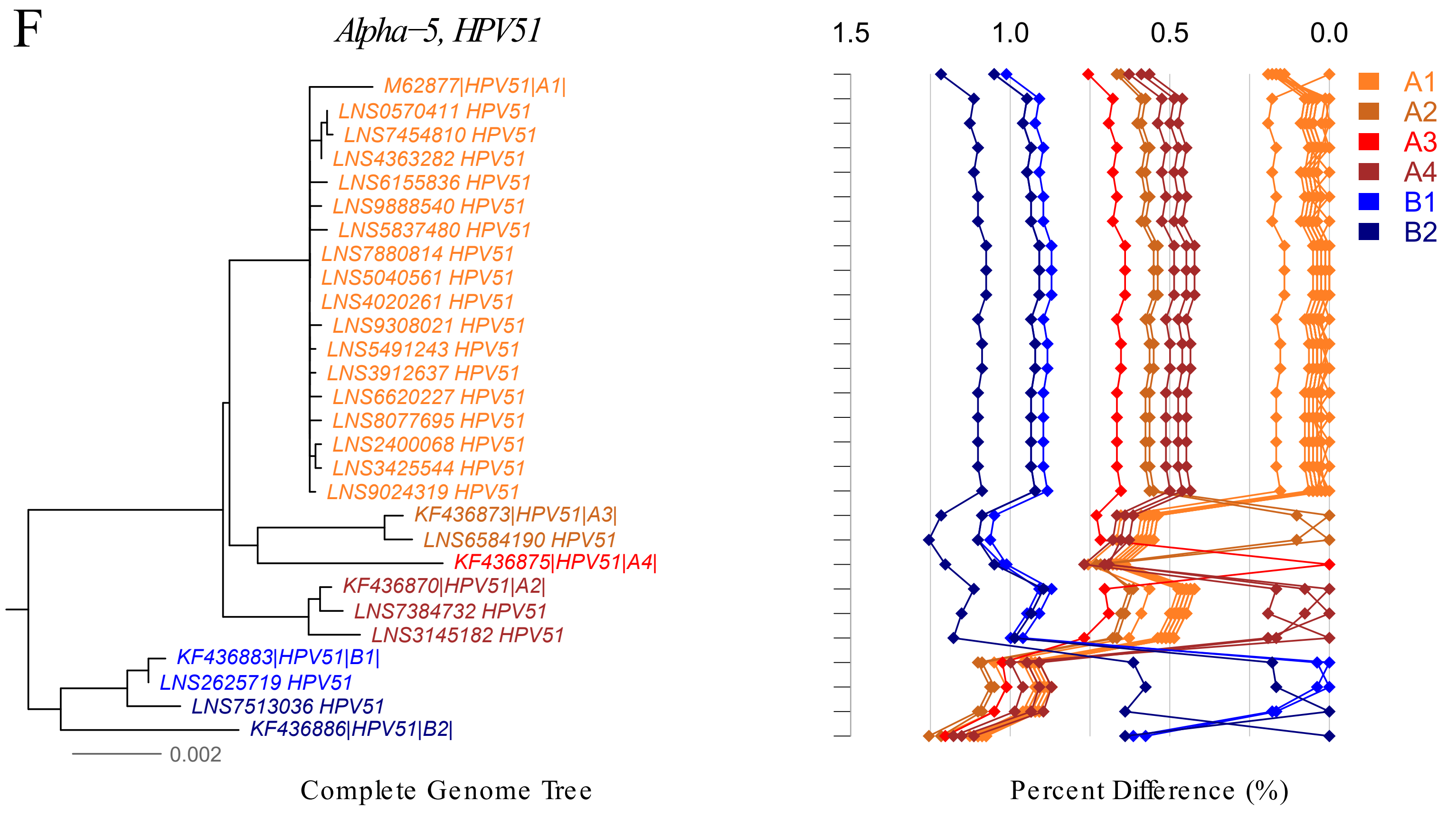
F
Alpha-5, HPV51
M62877|HPV51|A1|
LNS0570411 HPV51
LNS7454810 HPV51
LNS4363282 HPV51
LNS6155836 HPV51
LNS9888540 HPV51
LNS5837480 HPV51
LNS7880814 HPV51
LNS5040561 HPV51
LNS4020261 HPV51
LNS9308021 HPV51
LNS5491243 HPV51
LNS3912637 HPV51
LNS6620227 HPV51
LNS8077695 HPV51
LNS2400068 HPV51
LNS3425544 HPV51
LNS9024319 HPV51
KF436873|HPV51|A3|
LNS6584190 HPV51
KF436875|HPV51|A4|
KF436870|HPV51|A2|
LNS7384732 HPV51
LNS3145182 HPV51
KF436883|HPV51|B1|
LNS2625719 HPV51
LNS7513036 HPV51
KF436886|HPV51|B2|
0.002
Complete Genome Tree
1.5
1.0
0.5
0.0
A1
A2
A3
A4
B1
B2
Percent Difference (%)

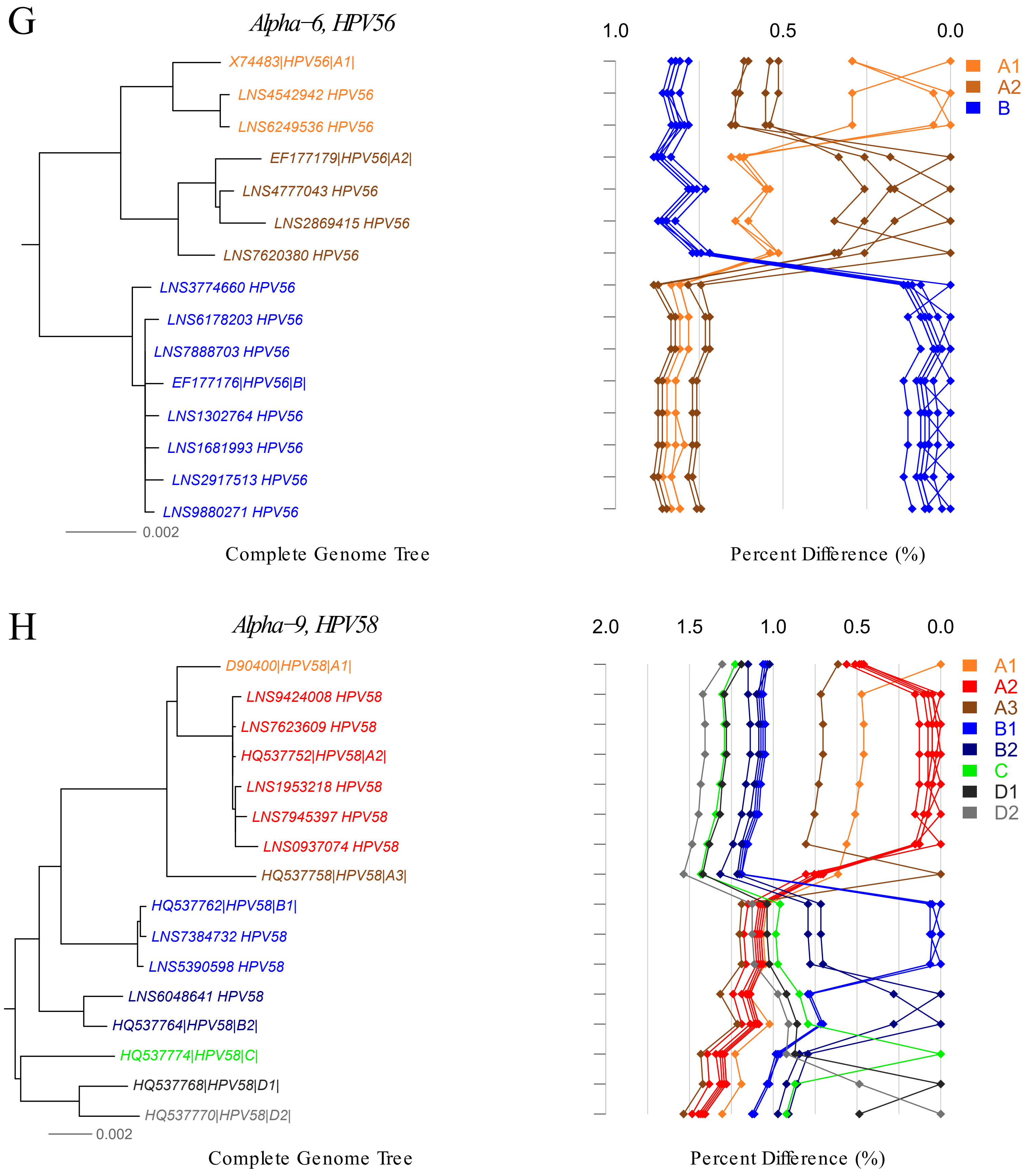
G
Alpha-6, HPV56
X74483|HPV56|A1|
LNS4542942 HPV56
LNS6249536 HPV56
EF177179|HPV56|A2|
LNS4777043 HPV56
LNS2869415 HPV56
LNS7620380 HPV56
LNS3774660 HPV56
LNS6178203 HPV56
LNS7888703 HPV56
EF177176|HPV56|B|
LNS1302764 HPV56
LNS1681993 HPV56
LNS2917513 HPV56
LNS9880271 HPV56
0.002
Complete Genome Tree
1.0
0.5
0.0
A1
A2
B
Percent Difference (%)
H
Alpha-9, HPV58
D90400|HPV58|A1|
LNS9424008 HPV58
LNS7623609 HPV58
HQ537752|HPV58|A2|
LNS1953218 HPV58
LNS7945397 HPV58
LNS0937074 HPV58
HQ537758|HPV58|A3|
HQ537762|HPV58|B1|
LNS7384732 HPV58
LNS5390598 HPV58
LNS6048641 HPV58
HQ537764|HPV58|B2|
HQ537774|HPV58|C|
HQ537768|HPV58|D1|
HQ537770|HPV58|D2|
0.002
Complete Genome Tree
2.0
1.5
1.0
0.5
0.0
A1
A2
A3
B1
B2
C
D1
D2
Percent Difference (%)

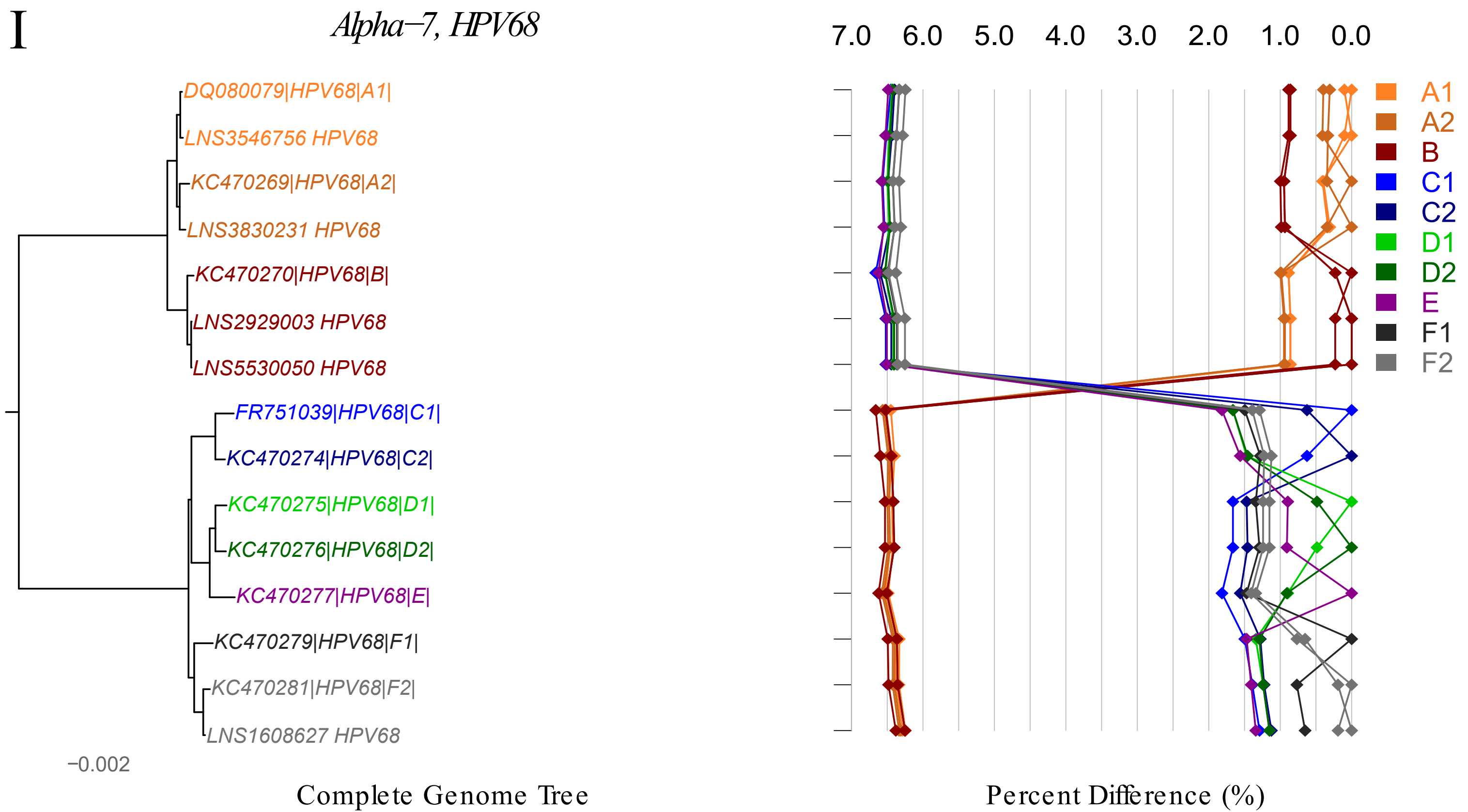
I
Alpha-7, HPV68
DQ080079|HPV68|A1|
LNS3546756 HPV68
KC470269|HPV68|A2|
LNS3830231 HPV68
KC470270|HPV68|B|
LNS2929003 HPV68
LNS5530050 HPV68
FR751039|HPV68|C1|
KC470274|HPV68|C2|
KC470275|HPV68|D1|
KC470276|HPV68|D2|
KC470277|HPV68|E|
KC470279|HPV68|F1|
KC470281|HPV68|F2|
LNS1608627 HPV68
−0.002
Complete Genome Tree
7.0
6.0
5.0
4.0
3.0
2.0
1.0
0.0
A1
A2
B
C1
C2
D1
D2
E
F1
F2
Percent Difference (%)
