# Supplementary material for: Characterization and Diversity of 243 Complete Human Papillomavirus Genomes in Cervical Swabs Using Next Generation Sequencing
Source: Viruses. 2020 Dec 14;12(12):1437. doi: 10.3390/v12121437 (PMC7764970; doi:10.3390/v12121437)
Supplement: Supplementary file 1 [file viruses-12-01437-s001.zip › Supplementary material/Supplementary Information.docx]

Characterisation and Diversity of 243 Complete Human Papillomavirus Genomes in Cervical Swabs Using Next Generation Sequencing

**Supplemental Material**

**Next generation sequencing**

**Library preparation**

Total DNA extracts were enriched using rolling-circle amplification (RCA) technology with the TempliPhi 100 kit according to manufacturer’s instructions (GE Healthcare Life Sciences, New Jersey, USA) [1]. Libraries were prepared using Nextera XT DNA Library Prep Kits (Illumina Inc., San Diego, USA) as recommended by manufacturer’s instructions, followed by sequencing on Illumina Miniseq Platform (Illumina Inc., San Diego, USA). Up to 96 samples were multiplexed using Nextera DNA CD Indexes in one run.

**Bioinformatic analysis**

**Full genome Assembly**

In order to assemble full HPV genome sequences, we followed two complementary approaches.

The first approach was to assemble all reads from all Papillux samples (N=744) remaining after filtering for human, bacterial, plasmid and fungal reference sequences, further on referred as “blind assembly”. The second approach (further on referred as “bowtie assembly”) is a reference based assembly, which was done on read collections resulting from filtering out human sequences and pre-mapping the remaining ones to the HPV reference genome sequences.

**Blind Assembly of full genomes.**

Raw reads were exposed to an initial FastQC run followed by visual inspection of FastQC report for each sample [2]. Assuming that identical paired read sequences are rather due to PCR amplification bias than real sequencing results, paired read data have been deduplicated (inhouse-python-script). Next, we applied a 2-step trimming using Trimmomatic (v. 0.38) [3]. The first step trims in favor of read correctness (MAXINFO:50:0.8 MINLEN:50), the second step represents an additional quality filter (SLIDINGWINDOW:5:20 MINLEN:50). The minimum read length accepted after trimming is 50bp. Single reads, resulting from disrupted read pairs, have been kept in the dataset. We re-run FastQC on trimmed samples to confirm good read quality profile by visual inspection of the FastQC reports. Samples were filtered from “contaminants” as follows : 1) end-to-end alignment of reads against the human reference genome (GRCh38) with Bowtie2 (version 2.3.5.1) in paired end mode for paired data and single end mode for single reads (single read data remained after trimming); 2) end-to-end alignment of remaining reads against all complete bacterial genome assemblies provided by GenBank (only “Complete Genome” assemblies are extracted from ftp://ftp.ncbi.nlm.nih.gov/genomes/genbank/bacteria/assembly_summary.txt, Sept 2018, N=11448) with Bowtie2 in single end mode only; 3) end-to-end alignment of remaining reads against all refseq-plasmid sequences (taken Nov 2018, ftp://ftp.ncbi.nlm.nih.gov/genomes/refseq/plasmid/plasmid.*.*.genomic.fna.gz, N=13924) with Bowtie2 in single end mode only and 4) end-to-end alignment of remaining reads against all complete fungal genome assemblies provided by GenBank (only “Complete Genome” assemblies are extracted from ftp://ftp.ncbi.nlm.nih.gov/genomes/genbank/fungi/assembly_summary.txt, Nov 2018, , N=40) with Bowtie2 in single end mode only [4].

The resulting fastq-files have been submitted to SPAdes (v. 3.13.0) [5] for *de novo* with the parameters --only-assembler –careful. Next, we selected for each sample only contigs longer or equal to a length of 1000bp and performed a BLAST (blastn, v. 2.9.0+) search against blast database holding 1.156.555 viral sequences downloaded from NCBI Virus (<https://www.ncbi.nlm.nih.gov/labs/virus>, January 2020).[6]. The blast output was pre-processed in order to find the best mapping reference sequence (best blast hit(s) based on the blast score) per contig in order to build blast based mapping genome assemblies. In case of several contigs per reference, we merged the contigs either by sequence mapping overlap or by filling gaps with Ns. We applied a re-blast of the generated genome assembly to our reference database and if the resulting output was represented by a single mapping covering the full length of the reference sequence, we considered the assembly as a complete genome. Genomes containing at least 0.2% of Ns were resolved either by remapping of reads or by alignments with respective reference genomes.

**Bowtie Assembly of full genomes**

Starting with raw data (N=744), we performed a rather stringend QC, to keep only high quality paired end reads in the assembly dataset. We made a first visual inspection of FastQC output, followed by deduplication of paired end read data, assuming that absolutely identical read paired derive from PCR amplification bias. Next we performed a 2-step trimming step with Trimmomatic (v. 0.38) (1. round parameters: PE, MAXINFO:50:0.8 MINLEN:100; 2. round parameters: SLIDINGWINDOW:5:20 MINLEN:100) [3], followed by FastQC to visually inspect and confirm good quality of remaining read pairs. Read pairs which have been disrupted due to low quality of one mate were discarded. After, data have been cleaned from human contamination my mapping read pairs to the human reference genome (GRCh38) with Bowtie2 suppressing unpaired alignments (parameters used: -X 1500, --no-mixed) [4]. To retrieve read pairs mapping to any HPV genotype, we set up a reference sequence set of 319 HPV genotype sequences (deduced from PAVE and one inhouse detected genotype not published, Sept 2018 (Supplementary Table )) [7]. Due to the circular genome of HPV and an expected lack of sufficient mapping in the start and end regions of a linearized genome sequence, we added the last 150bp (maximum read length) of the linearized genomes at the beginning and the first 150bp of the linearized genomes to the end of the reference sequences. From this modified reference sequence set, we built a bowtie2 index. Mapping has been done in paired-end mode, allowing an insert size of 8300bp (modified genome size of HPV), suppressing unpaired and discordant alignments, only reporting concordant but multiple mappings (bowtie2 parameters: -X 8300, -a, --no-mixed, --no-discordant, --al-conc) [4].

As Bowtie2 was set to report all mapping (meaning also multiple mappings of pairs to different references), a post filtering was done, to assure that only the best mapping concerning the different reference genomes is kept in the downstream analysis. Therefore, each read pair was assigned to exactly one reference, namely the one with the lowest mismatch count (counting insertions and deletion also as single mismatch per position). In case of equally good mappings to different references, the pair is assigned as “ambiguous” and not present in subsequent analysis steps. Next, we extracted a set of HPV positive samples (N=337) and re-created for each HPV genotype a fastq-file, based on respective sequence alignment mapping files. We corrected for 150bp flanking sequences being added before HPV reference sequence mapping. In case read mapping coordinates were located within these flanking regions, we spitted them accordingly, applying a minimum fragment length of 10bp. In order to select samples showing a high coverage of HPV genome, as major requirement for high quality full genome assembly, we applied a remapping of the corrected fastq-files with Bowtie2 only to the respective HPV genotype. From these mapping files we calculated the coverage of the respective HPV genotype genome with bedtools (v2.26.0) [8]. If the coverage of the respective HPV genotype genome was greater/equals to 99% we submitted the corrected fastq-files to SPAdes for *de novo* assembly (parameters -s, --only-assembler –careful) [5]. Next, we blasted all generated scaffolds against an inhouse-compiled blast database holding 2262 HPV sequences extracted from NCBI/Nucleotide (August 2019), covering also different HPV lineages and subtypes, providing therefore a higher resolution than HPV reference genomes only. Then we processed the blast output in order to find the best mapping reference sequence (best blast hit(s) based on the blast score) per contig and build blast based mappings genome assemblies. In case of several contigs per reference, we merged the contigs either by sequence mapping overlap or by filling gaps with Ns. During this merging procedure, we apply a re-blast of the generated genome assembly to our reference database to ensure that the constructed assembly is correct.

**Selection of full genome assemblies**

In case of HPV genomes assembled by both methods, we compared the sequences aiming to select the better one. We performed a Needleman-Wunsch global alignment for each HPV genome assembled by both approaches (needle tool, EMBOSS v6.6.0.0) [9]. In case the assembled genomes sequences are absolutely identical, we kept the Bowtie2-mapping based. We obtained 75 identical genomes by both methods, 86 not fully identical, 42 only with blind assembly and 43 only with bowtie assembly. For not fully identical genomes, but fund by both methods, we set up a pipeline generating data to support a genome selection based on expert knowledge and educated guess.

The pipeline comprised the following steps: a) creating a modified version for each genome assembly accounting for the circularity of the HPV genome as described before and build a bowtie2 index; b) re-map fastq-files for each sample (input fastq-files as used for blind assembly) against the modified genome assembly with bowtie2 in single-end mode; c) filter out unmapped reads; d) extract from mapping data the mapping read count for each assembly, the mismatch-count of each assembly (counting INS and DEL as single event), the number of reads specifically mapping only to one assembly but not to the one generated with the other method e) create a fastq-file correcting for 150bp-flanking sequences added in step a) and apply a bowtie2 mapping against the original (linearized) genome assembly to generate a mapping image by means of IGV in batch mode to visually inspect the read mapping; f) perform a blast search against the nucleotide collection blast database nt (Partially non-redundant nucleotide sequences from all traditional divisions of GenBank, EMBL, and DDBJ excluding GSS,STS, PAT, EST, HTG, and WGS, downloaded Dec 2019 from https://ftp.ncbi.nlm.nih.gov/blast/db/v5/), filter out the best mapping based on blast score and extract mapping measures like alignment length, mismatchcount or gapcount, calculate percentage of assembly sequence covered by alignment and the number of splits blast did to align the assembly sequence against the best reference found; g) create and visually inspect an alignment of assemblies with respective reference genome using MAFFT 1.4.0 [10].

For those assemblies only found by the one or the other method, we applied the procedure as described above covering steps a) to e) to see how well the assembly is represented by raw read data. Genomes with less than 0.2% of Ns, which were resolved either by remapping of reads or by alignments with respective reference genomes.

To assure that all our assemblies represent distinct genotypes, we investigated whether reads were mapping to several assemblies, considering all combinations of all generated assemblies (blind, bowtie) per a sample. Six samples were identified with more than 20 reads mapping to more than one assembly (data not shown). These reads were assigned to the assembly they mapped better or were removed from all concerned bam-files (based on the NM-Tag in the concerned bam-files). Then, we manually checked remapping results to assembled genomes using IGV and Geneious ([https://www.geneious.com](http://www.geneious.com/)). Moreover, we also manually checked alignments to the reference sequences and remapped reads for all assemblies.

Three HPV genomes (HPV42, HPV53 and HPV56) were removed from further analysis due to chimeric artefacts.

**Annotation of selected HPV genomes**

To annotate the selected genomes we applied VAPiD - a python implementation of for Viral Annotation Pipeline and iDentification [11]. We adapted the code to serve our needs with regard to genbank-file format (e.g. title, sequence type). We created a self-compiled reference blast database based on ~ 34,000 HPV related sequences, including 2,670 full HPV genome sequences. We assessed the completeness of annotation by investigating the genes which have been annotated.

**HPV detection**

Indexing tags from the Miniseq were used to automatically assign sequence reads to samples. We started bioinformatics analysis with initial quality control followed by visual inspection to get a first impression of the data. Duplicate reads were removed with an in-house python script. Low quality reads were trimmed according to the Phred quality score (minimum mean Phred quality score of 5 bases below 20) using Trimmomatic (v. 0.38) followed by quality checking and visual inspection. Reads with length below 100bp were removed [3]. Trimmed reads were filtered against the human genome (GRCh38) downloaded from GenBank (November 2018) using Bowtie2 with end-to-end alignment and paired end mode [4]. For HPV detection and genotyping, remaining reads were mapped with Bowtie2 to the reference set of 318 HPV sequences downloaded from PaVE (November 2018) and one novel genotype detected in our laboratory [7, 12].

Samples were considered positive, if at least 1 paired read was detected covering a minimum of 150bp of the reference genome. To avoid possible mapping artefacts reads covering <200pb with the mutation rate >0.03 (~4.5 variants on 150bp read) were blasted against NCBI based database (build on July 2019) and visually inspected. If the read exclusively mapped to the same genome as using bowtie2, artefact-status have been removed. All analyses were performed using in-house R (www.R-project.org/), python (www.python.org) and bash (http://www.gnu.org/software/bash/) scripts running on a Linux environment.

**Phylogenetic analysis and variant classificataion**

Complete HPV variants genomes obtained in this study (n=243) were assessed to investigate HPV variant distribution in healthy women in Luxembourg. Complete genomes were linearized according to the respective reference sequence available in PaVE [7] and aligned using MAFFT with default parameters [13]. Each HPV isolate genome was classified into lineage or sublineage using the p-distance method and phylogenetic analysis with respective reference [14-16]. For the phylogenetic analysis the evolutionary history was inferred using RAxML method employing 1,000 bootstrap values (raxmlHPC -f a -m GTRGAMMA -p 12345 -x 12345 -# 1000 –s allignment.fasta -n Tree) [17]. P-distance calculation was done in MEGA7 [18]. For HPV genomes with well-established lineages/sublineages, we used the respective reference lineages and sublineages in PaVE and Chen et al. [15], whereas for HPVs with no established lineages/sublineages, all complete genomes available in NCBI were downloaded for analysis (Supplemental Table S3). Phylogenetic trees were visualized in iTOL5.3 and Dendroscope 3 [19, 20]. Plots were constructed in R3.3. (scripts available on request).

Scripts are available from the authors on request.

**Supplementary results**

**Complete genome recovery and cytology**

In total, 74 (30.5%) of 243 complete genomes were obtained from women with low-grade squamous intraepithelial lesions (LSIL), 24 (9.9%) from women with atypical squamous cells of undetermined significance (ASC-US) and 144 (59.3%) from women with negative cytology. We recovered at least one complete genome from 52/67 (77.6%) of women with LSIL+ and 100/660 (15.1%) of women with negative cytology (Supplemental Table S6).

**References:**

1. Rector A, Tachezy R, Van Ranst M. A sequence-independent strategy for detection and cloning of circular DNA virus genomes by using multiply primed rolling-circle amplification. J Virol. 2004;78(10):4993-8.

2. Andrews S. (2010). FastQC: a quality control tool for high throughput sequence data. Available online at: <http://www.bioinformatics.babraham.ac.uk/projects/fastqc>.

3. Bolger AM, Lohse M, Usadel B. Trimmomatic: a flexible trimmer for Illumina sequence data. Bioinformatics (Oxford, England). 2014;30(15):2114-20.

4. Langmead B, Salzberg SL. Fast gapped-read alignment with Bowtie 2. Nat Methods. 2012;9(4):357-9.

5. Bankevich A, Nurk S, Antipov D, Gurevich AA, Dvorkin M, Kulikov AS, et al. SPAdes: a new genome assembly algorithm and its applications to single-cell sequencing. J Comput Biol. 2012;19(5):455-77.

6. Altschul SF, Gish W, Miller W, Myers EW, Lipman DJ. Basic local alignment search tool. J Mol Biol. 1990;215(3):403-10.

7. Van Doorslaer K, Li Z, Xirasagar S, Maes P, Kaminsky D, Liou D, et al. The Papillomavirus Episteme: a major update to the papillomavirus sequence database. Nucleic Acids Res. 2017;45(D1):D499-D506.

8. Quinlan AR, Hall IM. BEDTools: a flexible suite of utilities for comparing genomic features. Bioinformatics (Oxford, England). 2010;26(6):841-2.

9. Needleman SB, Wunsch CD. A general method applicable to the search for similarities in the amino acid sequence of two proteins. Journal of Molecular Biology. 1970;48(3):443-53.

10. Katoh K, Misawa K, Kuma K, Miyata T. MAFFT: a novel method for rapid multiple sequence alignment based on fast Fourier transform. Nucleic Acids Res. 2002;30(14):3059-66.

11. Shean RC, Makhsous N, Stoddard GD, Lin MJ, Greninger AL. VAPiD: a lightweight cross-platform viral annotation pipeline and identification tool to facilitate virus genome submissions to NCBI GenBank. BMC Bioinformatics. 2019;20(1):48.

12. Arroyo LS, Smelov V, Bzhalava D, Eklund C, Hultin E, Dillner J. Next generation sequencing for human papillomavirus genotyping. J Clin Virol. 2013;58(2):437-42.

13. Katoh K, Standley DM. MAFFT multiple sequence alignment software version 7: improvements in performance and usability. Mol Biol Evol. 2013;30(4):772-80.

14. Chen Z, Schiffman M, Herrero R, Desalle R, Anastos K, Segondy M, et al. Evolution and taxonomic classification of human papillomavirus 16 (HPV16)-related variant genomes: HPV31, HPV33, HPV35, HPV52, HPV58 and HPV67. PLoS One. 2011;6(5):e20183.

15. Chen Z, Schiffman M, Herrero R, DeSalle R, Anastos K, Segondy M, et al. Classification and evolution of human papillomavirus genome variants: Alpha-5 (HPV26, 51, 69, 82), Alpha-6 (HPV30, 53, 56, 66), Alpha-11 (HPV34, 73), Alpha-13 (HPV54) and Alpha-3 (HPV61). Virology. 2018;516:86-101.

16. Chan PK, Zhang C, Park JS, Smith-McCune KK, Palefsky JM, Giovannelli L, et al. Geographical distribution and oncogenic risk association of human papillomavirus type 58 E6 and E7 sequence variations. Int J Cancer. 2013;132(11):2528-36.

17. Stamatakis A. RAxML version 8: a tool for phylogenetic analysis and post-analysis of large phylogenies. Bioinformatics. 2014;30(9):1312-3.

18. Kumar S, Stecher G, Tamura K. MEGA7: Molecular Evolutionary Genetics Analysis Version 7.0 for Bigger Datasets. Mol Biol Evol. 2016;33(7):1870-4.

19. Letunic I, Bork P. Interactive Tree Of Life (iTOL) v4: recent updates and new developments. Nucleic Acids Res. 2019;47(W1):W256-W9.

20. Huson DH, Scornavacca C. Dendroscope 3: an interactive tool for rooted phylogenetic trees and networks. Syst Biol. 2012;61(6):1061-7.
