# Supplementary material for: Characterization and Diversity of 243 Complete Human Papillomavirus Genomes in Cervical Swabs Using Next Generation Sequencing
Source: Viruses. 2020 Dec 14;12(12):1437. doi: 10.3390/v12121437 (PMC7764970; doi:10.3390/v12121437)
Supplement: Supplementary file 1 [file viruses-12-01437-s001.zip › Supplementary material/Supplementary Table S4.docx]

Supplementary Table S4. Genotypes specific agreement between Anyplex and NGS.

| High risk | Genotype | A-/NGS-^1^ | A+/NGS-^2^ | A-/NGS+^3^ | A+/NGS+^4^ | % agreement | Kappa (se) | Interpretation^5^ |
| --- | --- | --- | --- | --- | --- | --- | --- | --- |
|  | 16 | 699 | 13 | 8 | 9 | 97.1 | 0.447 (0.037) | M |
|  | 18 | 724 | 2 | 0 | 3 | 99.7 | 0.749 (0.036) | G |
|  | 31 | 696 | 5 | 9 | 19 | 93.1 | 0.721 (0.037) | G |
|  | 33 | 712 | 4 | 1 | 13 | 99.3 | 0.835 (0.037) | E |
|  | 35 | 718 | 3 | 0 | 8 | 99.6 | 0.840 (0.037) | E |
|  | 39 | 692 | 10 | 11 | 16 | 97.1 | 0.589 (0.037) | M |
|  | 45 | 719 | 2 | 2 | 6 | 99.5 | 0.747 (0.037) | G |
|  | 51 | 665 | 10 | 19 | 35 | 96.0 | 0.686 (0.037) | G |
|  | 52 | 681 | 15 | 14 | 19 | 96.0 | 0.546 (0.036) | M |
|  | 56 | 687 | 4 | 17 | 21 | 97.1 | 0.652 (0.036) | G |
|  | 58 | 679 | 26 | 6 | 18 | 95.6 | 0.509 (0.035) | M |
|  | 59 | 689 | 12 | 6 | 22 | 97.5 | 0.697 (0.037) | G |
|  | 66 | 681 | 15 | 9 | 24 | 96.7 | 0.650 (0.036) | G |
|  | 68 | 685 | 21 | 3 | 20 | 96.7 | 0.609 (0.034) | G |
| Low risk | 6 | 704 | 12 | 2 | 11 | 98.1 | 0.602 (0.036) | G |
|  | 11 | 728 | 1 | 0 | 0 | 99.9 | 0.000 (0.000) | P |
|  | 26 | 728 | 1 | 0 | 0 | 99.9 | 0.000 (0.000) | P |
|  | 40 | 701 | 13 | 3 | 12 | 97.8 | 0.589 (0.035) | M |
|  | 42 | 630 | 41 | 5 | 53 | 93.7 | 0.664 (0.035) | G |
|  | 43 | 700 | 15 | 1 | 13 | 97.8 | 0.609 (0.034) | G |
|  | 44 | 697 | 16 | 4 | 12 | 97.1 | 0.532 (0.035) | M |
|  | 53 | 630 | 52 | 7 | 40 | 91.9 | 0.536 (0.033) | M |
|  | 54 | 682 | 18 | 8 | 21 | 96.4 | 0.599 (0.035) | M |
|  | 61 | 701 | 9 | 2 | 17 | 98.5 | 0.748 (0.037) | G |
|  | 69 | 728 | 1 | 0 | 0 | 99.9 | 0.000 (0.000) | P |
|  | 70 | 708 | 9 | 2 | 10 | 98.5 | 0.638 (0.035) | G |
|  | 73 | 694 | 6 | 14 | 15 | 97.3 | 0.586 (0.037) | M |
|  | 82 | 717 | 0 | 7 | 5 | 99.0 | 0.584 (0.035) | M |

A, Anyplex; NGS, next generation sequencing; se, standard error.

^1^A-/NGS-, negative with both methods.

^2^A+/NGS-, Anyplex positive and NGS negative.

^3^A-/NGS+, Anyplex negative and NGS positive.

^4^A+/NGS+, positive with both methods.

^5^Interpretation of the kappa values. P, poor; F, fair; M, moderate; G, good; E, excellent.
