# Supplementary material for: Characterization and Diversity of 243 Complete Human Papillomavirus Genomes in Cervical Swabs Using Next Generation Sequencing
Source: Viruses. 2020 Dec 14;12(12):1437. doi: 10.3390/v12121437 (PMC7764970; doi:10.3390/v12121437)
Supplement: Supplementary file 1 [file viruses-12-01437-s001.zip › Supplementary material/Supplementary Table S7.docx]

Supplementary Table S7. Cytological abnormality and HPV positivity.

| Cytology | HPV positivity | | | |
| --- | --- | --- | --- | --- |
|  | Anyplex | | NGS | |
|  | Neg | Pos | Neg | Pos |
| HSIL | 0 (0.0) | 1 (100.0) | 0 (0.0) | 1 (100.0) |
| ASC-H | 1 (100.0) | 0 (0.0) | 1 (100.0) | 0 (0.0) |
| ASC-US | 3 (13.0) | 20 (87.0) | 2 (8.7) | 21 (91.3) |
| LSIL | 2 (4.8) | 40 (95.2) | 1 (1.4) | 41 (97.6) |
| NILM | 356 (53.9) | 304 (46.1) | 392 (59.4) | 268 (40.6) |
| Total | 362 (49.8) | 365 (50.21) | 396 (54.5) | 331 (45.5) |

Supplementary Table S6B. Cytological abnormality and complete genome recovery.

| Cytology | CG recovery | |  |  |  |
| --- | --- | --- | --- | --- | --- |
|  | Samples without CG | Samples with CG^1^ | Total | Total N (%) of CG | P-value^2^ |
| HSIL | 0 (0.0) | 1 (100.0) | 1 (0.1) | 1 (0.4) | 0.018 |
| ASC-H | 1(100.0) | 0 (0.0) | 1 (0.1) | 0 (0.0) | 0.673 |
| ASC-US | 9 (39.1) | 14 (60.9) | 23 (3.2) | 24 (9.9) | <0.001 |
| LSIL | 5 (11.9) | 37 (88.1) | 42 (5.8) | 74 (30.5) | <0.001 |
| LSIL+ | 15 (22.4) | 52 (77.6) | 67 (9.2) | 99 (40.7) | <0.001 |
| NILM | 560 (84.9) | 100 (15.1) | 660 (90.8) | 144 (59.3) |  |
| Total | 575 (79.1) | 152 (20.9) | 727 (100.0) | 243 (100.0) |  |

NILM, negative for intraepithelial lesions and malignancies; ASC-US, atypical squamous cells of undetermined significance; LSIL, low-grade squamous intraepithelial lesions; ASC-H, atypical squamous cells of undetermined significance, in which a high-grade squamous intraepithelial lesion cannot be excluded; HSIL, high-grade squamous intraepithelial lesions; CG, complete genome; NGS, next generation sequencing, Neg, negative.

^1^Samples with at least one complete genome recovered (range: 1-6).

^2^Chi2 test for comparison between abnormal cytology and NILM for complete genome recovery.
