# Supplementary material for: Characterization and Diversity of 243 Complete Human Papillomavirus Genomes in Cervical Swabs Using Next Generation Sequencing
Source: Viruses. 2020 Dec 14;12(12):1437. doi: 10.3390/v12121437 (PMC7764970; doi:10.3390/v12121437)
Supplement: Supplementary file 1 [file viruses-12-01437-s001.zip › Supplementary material/Supplementary Table S8.docx]

Supplementary Table S8. Overall agreement for HPV detection between Anyplex II HPV28 and next-generation sequencing with the detection threshold mapping coverage of 1000bp.

| HPV types^1^ | A+/NGS+^2^ | A+/NGS-^3^ | A-/NGS+^4^ | A-/NGS-^5^ | % agreement | Kappa (se) | Interp-retation^6^ |
| --- | --- | --- | --- | --- | --- | --- | --- |
| 28 HPV types^7^ | 239 | 127 | 28 | 335 | 78.7 | 0.575 (0.036) | G |
| 28 HPV types (++/+++)^8^ | 220 | 47 | 61 | 401 | 85.2 | 0.684 (0.037) | G |
| 14 HPV types^9^ | 150 | 89 | 32 | 447 | 83.4 | 0.611 (0.036) | G |
| 14 HPV types (++/+++)^10^ | 167 | 58 | 43 | 478 | 86.2 | 0.653 (0.037) | G |

A, Anyplex; NGS, next generation sequencing; se, standard error.

^1^Number of reads per sample restricted to 28 genotypes detectable by Anyplex

^2^A+/NGS+, positive with both methods.

^3^A+/NGS-, Anyplex positive and NGS negative.

^4^A-/NGS+, Anyplex negative and NGS positive.

^5^A-/NGS-, negative with both methods.

^6^Interpretation of the kappa values: P, poor; F, fair; M, moderate; G, good; E, excellent [47].

^7^Analysis restricted to 28 HPV types detectable by Anyplex.

^8^Analysis restricted to 28 HPV types detectable by Anyplex, Anyplex positivity is restricted to medium or high viral load (++/+++).

^9^Analysis is restricted to 14 high-risk HPV (HPV16,18,31,33,35,39,45,51,52,56,58,59, 66 and 68) [5].

^10^Analysis is restricted to 14 high-risk HPV, Anyplex positivity is restricted to viral load medium or high (++/+++).
