# Supplementary figures and images for: Characterization and Diversity of 243 Complete Human Papillomavirus Genomes in Cervical Swabs Using Next Generation Sequencing
Source: Viruses. 2020 Dec 14;12(12):1437. doi: 10.3390/v12121437 (PMC7764970; doi:10.3390/v12121437)

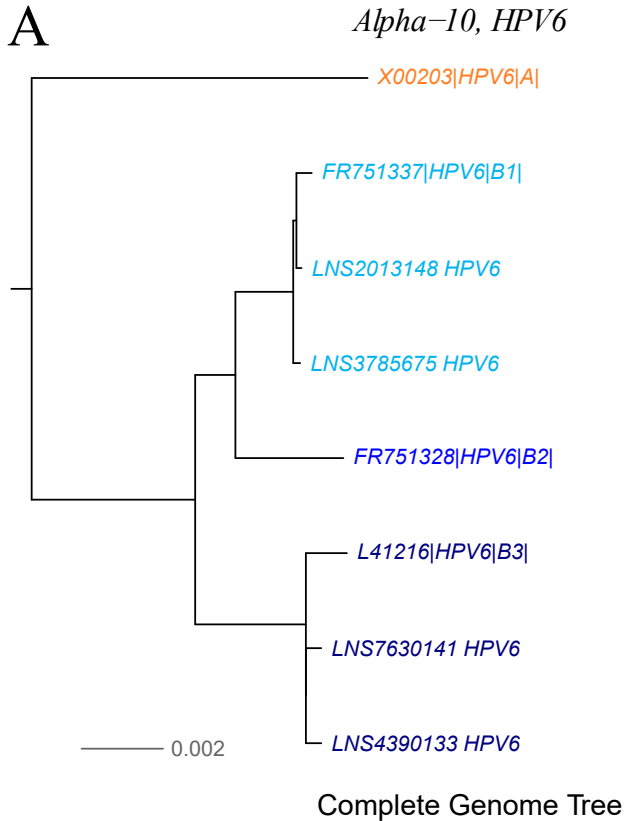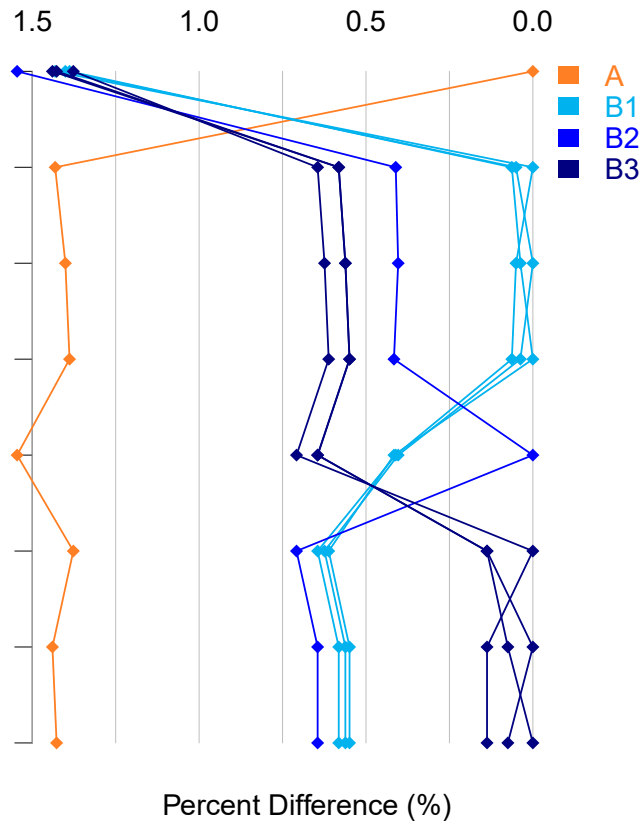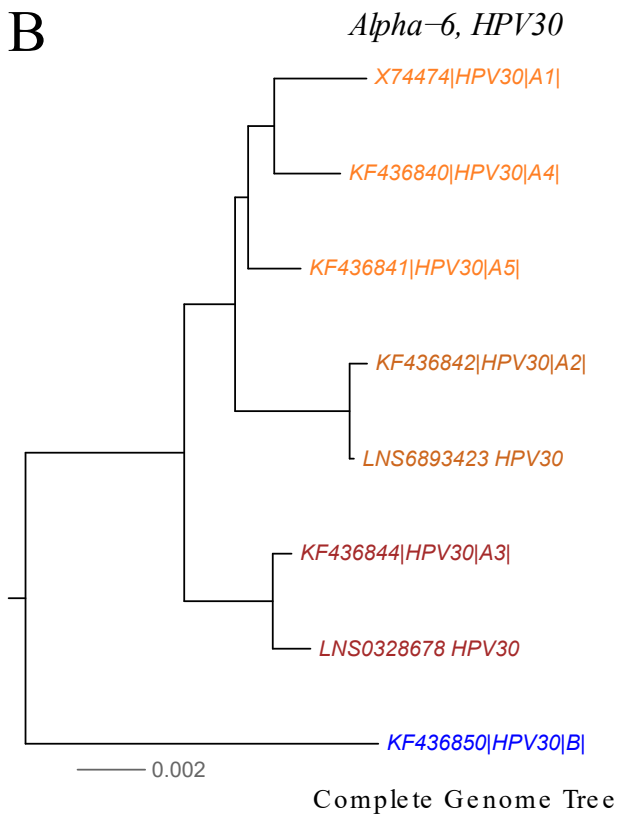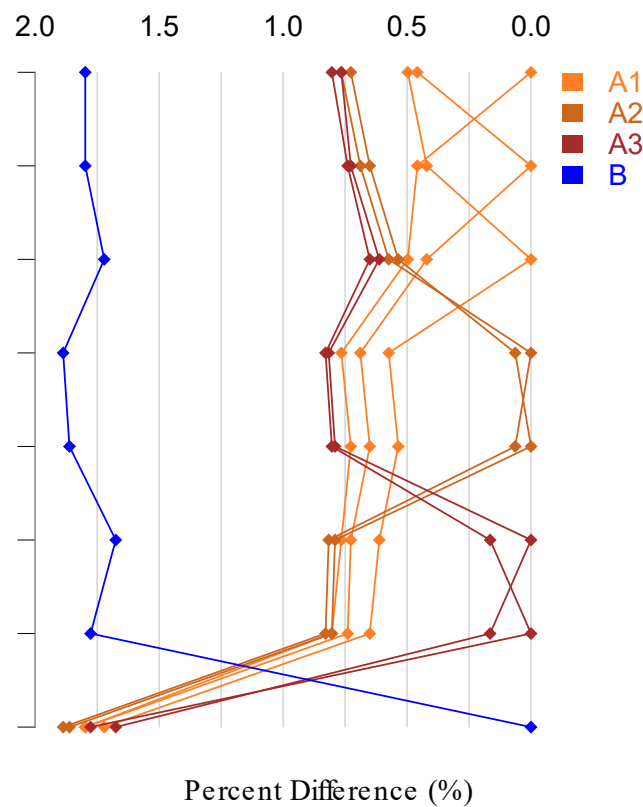

C

*Alpha-10, HPV44*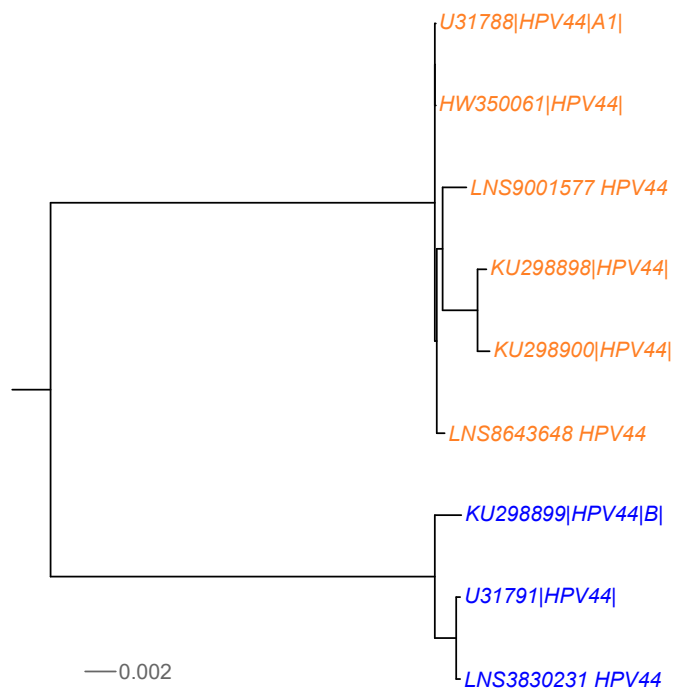

7.0 6.0 5.0 4.0 3.0 2.0 1.0 0.0

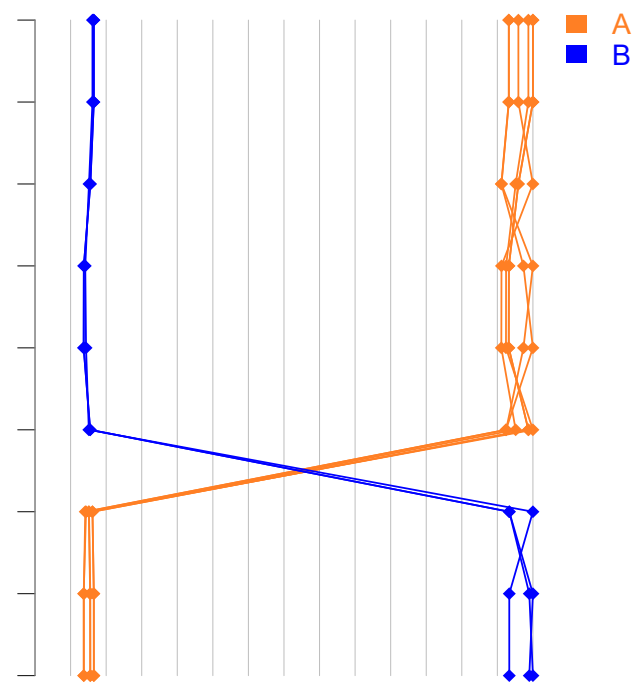

D

*Alpha-6, HPV53*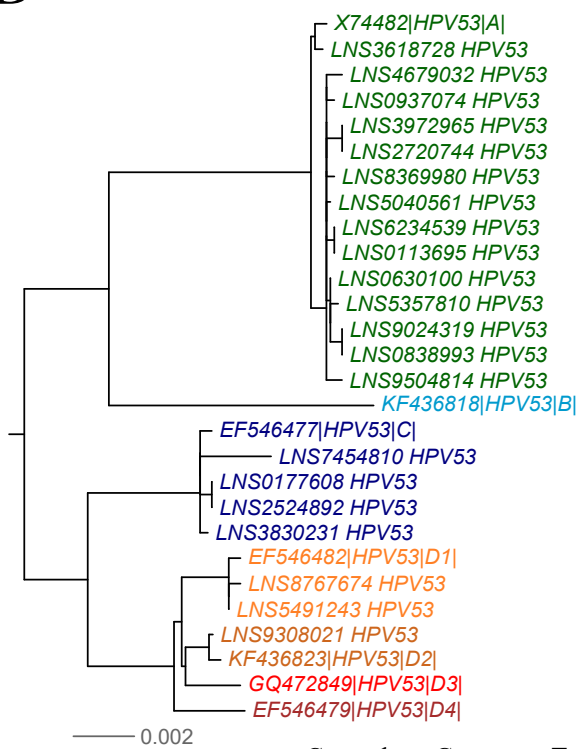

2.0 1.5 1.0 0.5 0.0

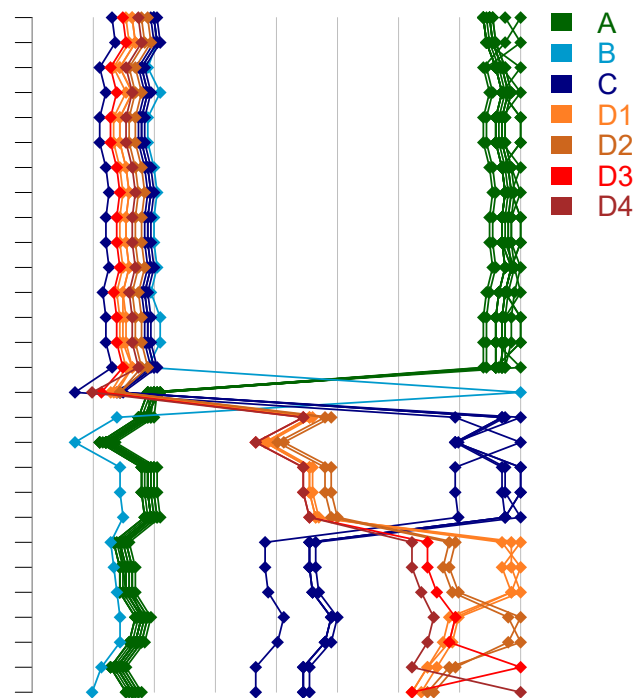

E

*Alpha-7, HPV70*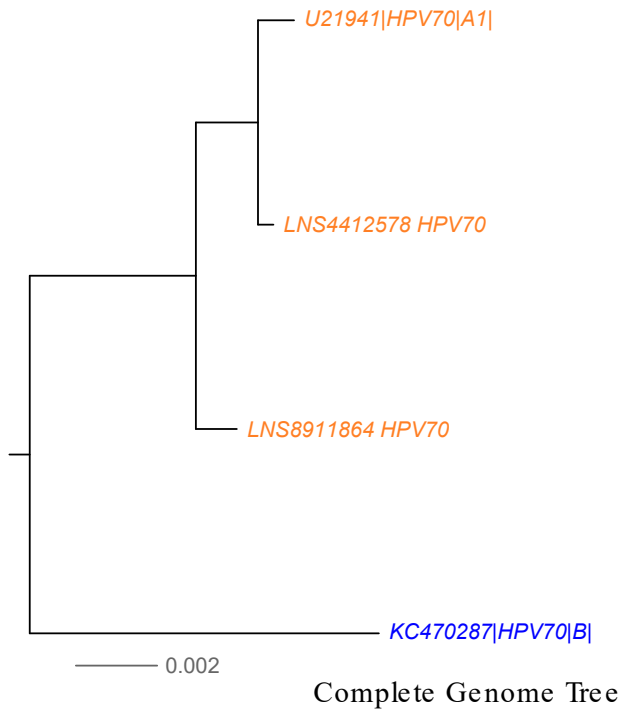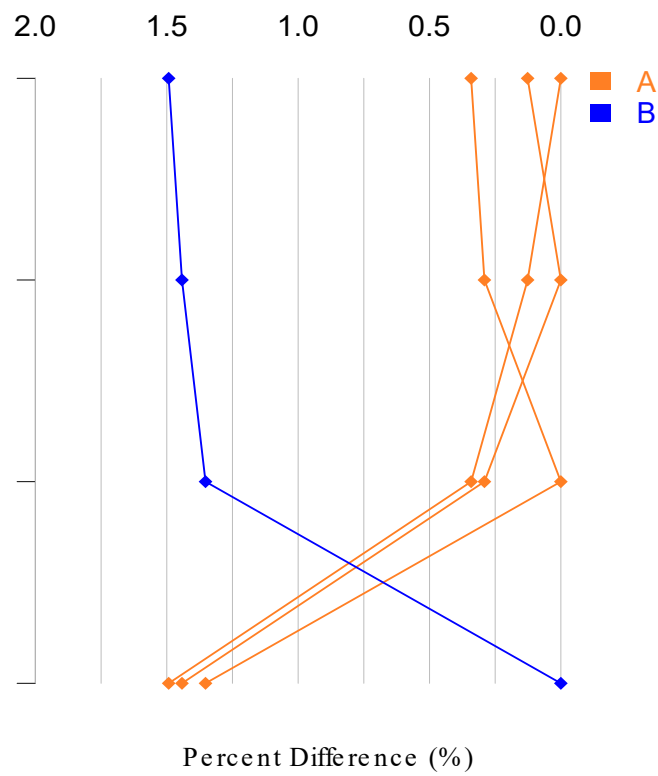

F

*Alpha-5, HPV82*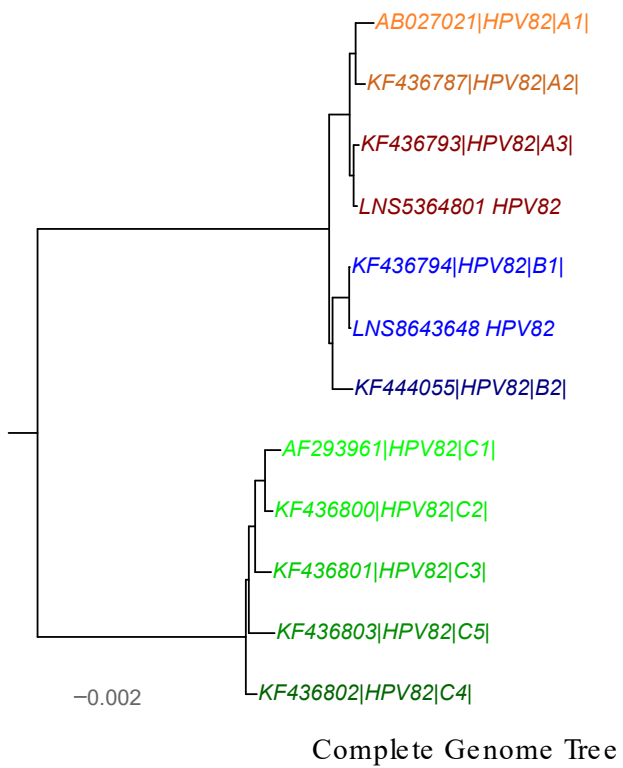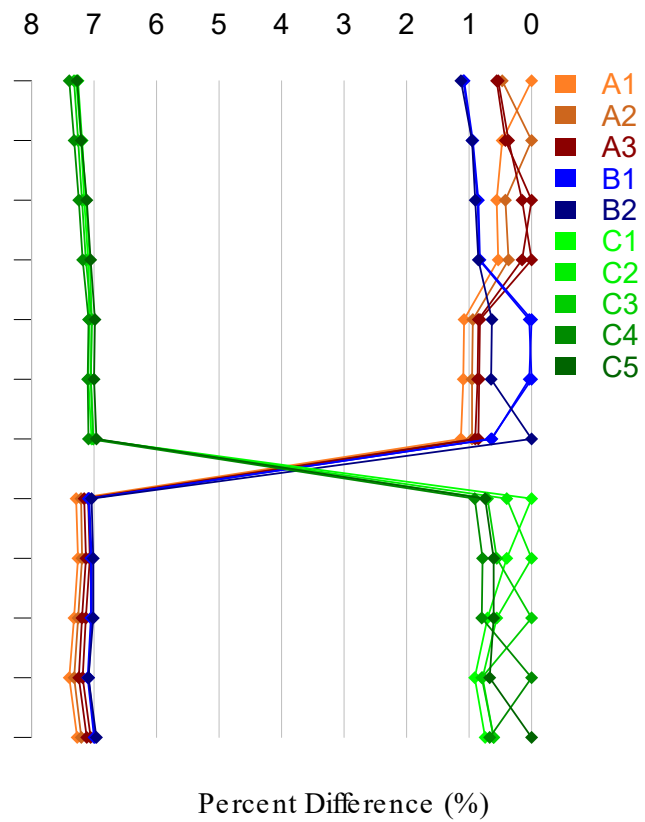

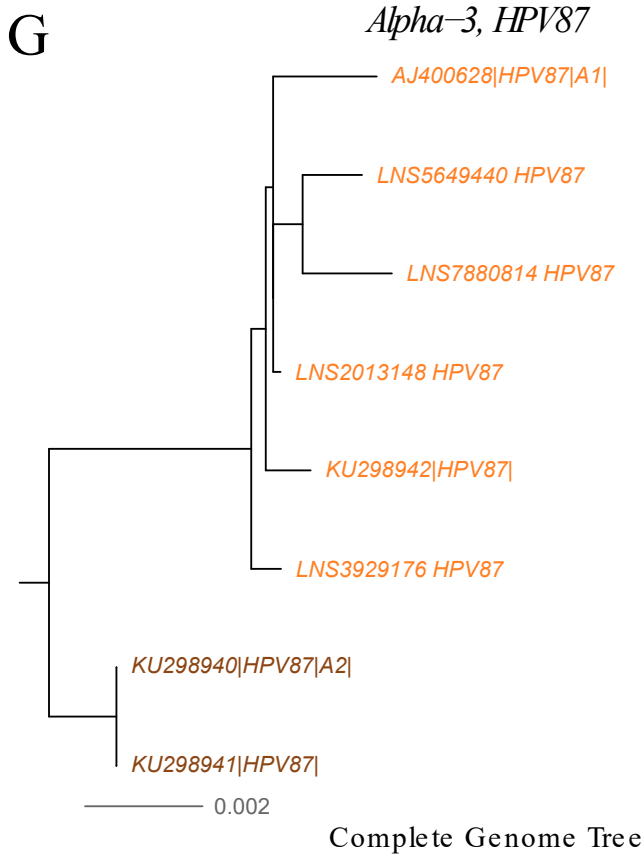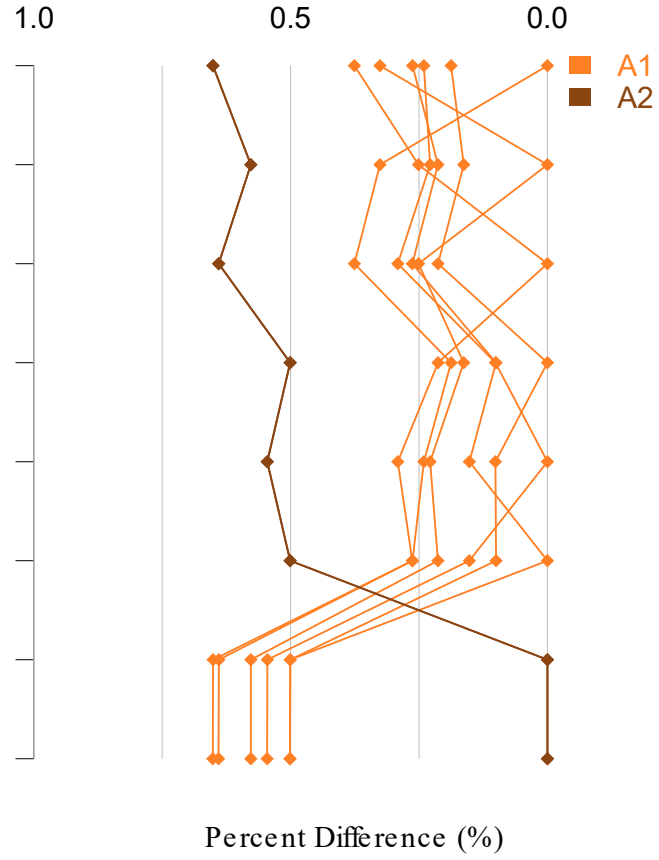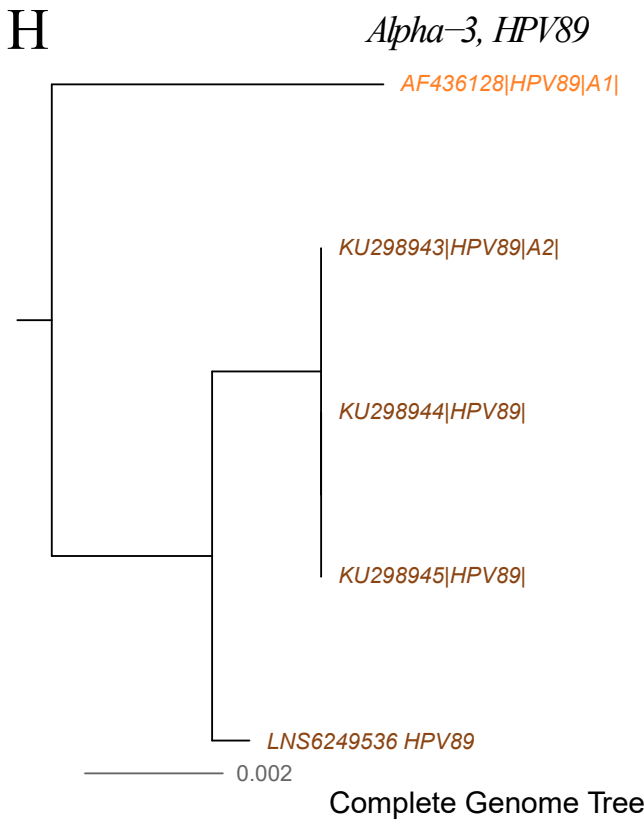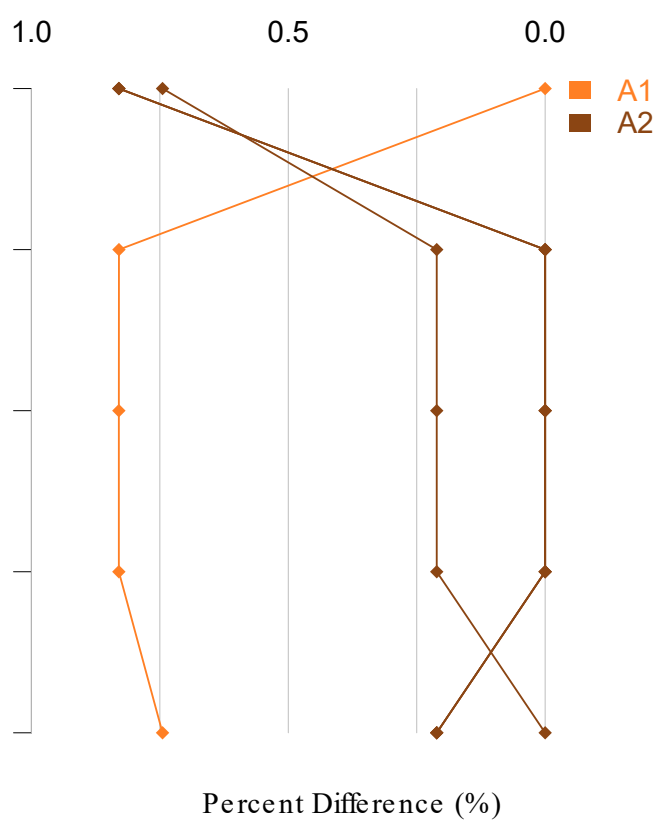

Supplement: Supplementary file 1 [file viruses-12-01437-s001.zip › Supplementary material/Supplementary Fig S4A-I.pdf]
